# Semantic Representations for NLP Using VerbNet and the Generative Lexicon

**DOI:** 10.3389/frai.2022.821697

**Published:** 2022-04-14

**Authors:** Susan Windisch Brown, Julia Bonn, Ghazaleh Kazeminejad, Annie Zaenen, James Pustejovsky, Martha Palmer

**Affiliations:** ^1^Department of Linguistics, University of Colorado Boulder, Boulder, CO, United States; ^2^Department of Linguistics, Stanford University, Stanford, CA, United States; ^3^Department of Computer Science, Brandeis University, Waltham, MA, United States

**Keywords:** semantics, natural language processing, lexical resource, semantic representation, lexicon, VerbNet, NLP

## Abstract

The need for deeper semantic processing of human language by our natural language processing systems is evidenced by their still-unreliable performance on inferencing tasks, even using deep learning techniques. These tasks require the detection of subtle interactions between participants in events, of sequencing of subevents that are often not explicitly mentioned, and of changes to various participants across an event. Human beings can perform this detection even when sparse lexical items are involved, suggesting that linguistic insights into these abilities could improve NLP performance. In this article, we describe new, hand-crafted semantic representations for the lexical resource VerbNet that draw heavily on the linguistic theories about subevent semantics in the Generative Lexicon (GL). VerbNet defines classes of verbs based on both their semantic and syntactic similarities, paying particular attention to shared diathesis alternations. For each class of verbs, VerbNet provides common semantic roles and typical syntactic patterns. For each syntactic pattern in a class, VerbNet defines a detailed semantic representation that traces the event participants from their initial states, through any changes and into their resulting states. The Generative Lexicon guided the structure of these representations. In GL, event structure has been integrated with dynamic semantic models in order to represent the attribute modified in the course of the event (the location of the moving entity, the extent of a created or destroyed entity, etc.) as a sequence of states related to time points or intervals. We applied that model to VerbNet semantic representations, using a class's semantic roles and a set of predicates defined across classes as components in each subevent. We will describe in detail the structure of these representations, the underlying theory that guides them, and the definition and use of the predicates. We will also evaluate the effectiveness of this resource for NLP by reviewing efforts to use the semantic representations in NLP tasks.

## 1. Introduction

The long-awaited time when we can communicate with computers naturally-that is, with subtle, creative human language-has not yet arrived. We've come far from the days when computers could only deal with human language in simple, highly constrained situations, such as leading a speaker through a phone tree or finding documents based on key words. We have bots that can write simple sports articles (Puduppully et al., 2019) and programs that will syntactically parse a sentence with very high accuracy (He and Choi, 2020). But question-answering systems still get poor results for questions that require drawing inferences from documents or interpreting figurative language. Just identifying the successive locations of an entity throughout an event described in a document is a difficult computational task.

Early rule-based systems that depended on linguistic knowledge showed promise in highly constrained domains and tasks. However, they could not scale to more general situations. Machine learning side-stepped the rules and made great progress on foundational NLP tasks such as syntactic parsing. When they hit a plateau, more linguistically oriented features were brought in to boost performance. Additional processing such as entity type recognition and semantic role labeling, based on linguistic theories, help considerably, but they require extensive and expensive annotation efforts. Deep learning left those linguistic features behind and has improved language processing and generation to a great extent. However, it falls short for phenomena involving lower frequency vocabulary or less common language constructions, as well as in domains without vast amounts of data. In terms of real language understanding, many have begun to question these systems' abilities to actually interpret meaning from language (Bender and Koller, 2020; Emerson, 2020b). Several studies have shown that neural networks with high performance on natural language inferencing tasks are actually exploiting spurious regularities in the data they are trained on rather than exhibiting understanding of the text. Once the data sets are corrected/expanded to include more representative language patterns, performance by these systems plummets (Glockner et al., 2018; Gururangan et al., 2018; McCoy et al., 2019).

There is a growing realization among NLP experts that observations of form alone, without grounding in the referents it represents, can never lead to true extraction of meaning-by humans or computers (Bender and Koller, 2020). One possible solution is to train systems on both language data and perceptual data (e.g., image data) (Bruni et al., 2014; Bulat et al., 2016), or by bringing in the attention-focusing means humans use to connect language to referents, such as gaze and gesture (Pustejovsky and Krishnaswamy, 2021). Another proposed solution-and one we hope to contribute to with our work-is to integrate logic or even explicit logical representations into distributional semantics and deep learning methods. Emerson has pursued learning logical representations instead of vectors (Emerson, 2020a), and has said, “I believe that the right approach is to learn a logically interpretable model, either by defining a vector space with logical structure or by directly using logical representations” (Emerson, 2020b, p. 7443).

With the goal of supplying a domain-independent, wide-coverage repository of logical representations, we have extensively revised the semantic representations in the lexical resource VerbNet (Dang et al., 1998; Kipper et al., 2000, 2006, 2008; Schuler, 2005). With its syntactically and semantically cohesive classes of verbs, VerbNet has long been used in NLP to improve such tasks as semantic role labeling, verb sense disambiguation and ontology mapping (Shi and Mihalcea, 2005; Giuglea and Moschitti, 2006; Loper et al., 2007; Brown et al., 2014; Indig et al., 2016).

Often compared to the lexical resources FrameNet and PropBank, which also provide semantic roles, VerbNet actually differs from these in several key ways, not least of which is its semantic representations. Both FrameNet and VerbNet group verbs semantically, although VerbNet takes into consideration the syntactic regularities of the verbs as well. Both resources define semantic roles for these verb groupings, with VerbNet roles being fewer, more coarse-grained, and restricted to central participants in the events. What we are most concerned with here is the representation of a class's (or frame's) semantics. In FrameNet, this is done with a prose description naming the semantic roles and their contribution to the frame. For example, the Ingestion frame is defined with “An **Ingestor** consumes food or drink **(Ingestibles)**, which entails putting the Ingestibles in the mouth for delivery to the digestive system. This may include the use of an **Instrument**.” VerbNet, however, uses first order logic representations with defined predicates to show the relationships between roles and to track any changes to the participants across the timeline of the event, with variations for each syntactic pattern included in the class.

VerbNet is also somewhat similar to PropBank and Abstract Meaning Representations (AMRs). PropBank defines semantic roles for individual verbs and eventive nouns, and these are used as a base for AMRs, which are semantic graphs for individual sentences. These representations show the relationships between arguments in a sentence, including peripheral roles like Time and Location, but do not make explicit any sequence of subevents or changes in participants across the timespan of the event. VerbNet's explicit subevent sequences allow the extraction of preconditions and postconditions for many of the verbs in the resource and the tracking of any changes to participants. In addition, VerbNet allow users to abstract away from individual verbs to more general categories of eventualities. We believe VerbNet is unique in its integration of semantic roles, syntactic patterns, and first-order-logic representations for wide-coverage classes of verbs.

VerbNet's semantic representations, however, have suffered from several deficiencies that have made them difficult to use in NLP applications. To unlock the potential in these representations, we have made them more expressive and more consistent across classes of verbs. We have grounded them in the linguistic theory of the Generative Lexicon (GL) (Pustejovsky, 1995, 2013; Pustejovsky and Moszkowicz, 2011), which provides a coherent structure for expressing the temporal and causal sequencing of subevents. Explicit pre- and post-conditions, aspectual information, and well-defined predicates all enable the tracking of an entity's state across a complex event.

In the rest of this article, we review the relevant background on Generative Lexicon (GL) and VerbNet, and explain our method for using GL's theory of subevent structure to improve VerbNet's semantic representations. We show examples of the resulting representations and explain the expressiveness of their components. Finally, we describe some recent studies that made use of the new representations to accomplish tasks in the area of computational semantics.

## 2. Motivation and Method

### 2.1. Classic VerbNet

VerbNet is a lexicon of around 5,200 English verbs, organized primarily around Levin (1993)'s verb classification. Classes in VerbNet are structured according to the verbs' syntactic behaviors, describing the diathesis alternations compatible with each verb (Bonial et al., 2011). Each VerbNet class contains semantic representations expressed as conjunctions of primitive predicates, such as **motion** or **cause**. Event participants that have syntactic relevance are identified with various stages of the event evoked by the syntactic frame. The original semantic representations, what we are here calling “Classic VerbNet,” were intended to provide *atomic* event representations associated with groups of semantically similar language predicates (i.e., English verbs). There are, however, several ways in which the Classic VerbNet semantics were unsatisfactory: (i) the representation was not first-order; (ii) there were no explicitly identified (reified) subevents that could be referenced in discourse models or planning algorithms; and (iii) there was no mention of the actual predicative change, i.e., the *opposition structure* that is implicated in a change-of-state event, such as *close* or *break*.

Consider the first issue. Classic VerbNet represented each event with a single event variable E, and predicates were temporally positioned relative to one another within the larger event through the inclusion of a second-order predicate, namely, **start**, **during**, or **end**. For example, the semantics for one intransitive frame in class **Run-51.3.2** is shown in (1)^1^.

(1)    Classic Verbnet representation
            *Billy ran into a cafe*.
            **path_rel**(start(E), Theme, ?Initial_location, ch_of_loc, prep)
            **motion**(during(E), Theme)
            **path_rel**(during(E), Theme, ?Trajectory, ch_of_loc, prep)
            **path_rel**(end(E), Theme, Destination, ch_of_loc, prep)

Since there was only a single event variable, any ordering or subinterval information needed to be performed as second-order operations. For example, temporal sequencing was indicated with the second-order predicates, **start**, **during**, and **end**, which were included as arguments of the appropriate first-order predicates.

However, as Zaenen et al. (2008) point out, the representation in (1) is still unable to support many temporal and spatial inferencing tasks, since the temporal ordering annotation was also not complete or consistent throughout the VerbNet database; for example, for several motion classes, **end**(E) was given but not **start**(E), and some classes involving change of location of participants (e.g., *gather, mix*) did not include a motion predicate at all. In order to accommodate such inferences, the event itself needs to have substructure, a topic we now turn to in the next section.

### 2.2. Generative Lexicon Event Structure

Many of the issues described in Section 2.1 can be resolved by adopting a linguistically motivated subevent representation for verb predication, such as that developed in Generative Lexicon Theory, where different Aktionsarten are distinguished in terms of their inherent subeventual structure (Pustejovsky, 1995). On this view, Vendler's “Aktionsarten” classes are associated with distinct event structures and their semantic interpretations (Vendler, 1967): state (b); process (c); achievement (d); and accomplishment (e). Subevents within an event are ordered by interval temporal relations (Allen, 1984), including: strict sequence, < _◦_ (Allen's “meet” relation); and strict overlap, ◦ (Allen's “identity” relation).

(2)    a. event→state ∣ process ∣ transition
       b. state: →   *e*
              **love**, **know**
       c. process: →   *e*_1_…*e*_*n*_
              **run**, **push**
       d. transition_*ach*_: → state state
              **open**, **die**
       e. transition_*acc*_: → process state
              **give**, **build**

In Im and Pustejovsky (2009); Im (2014), the basic logic of GL's event schema was applied to some VerbNet classes, to enrich the event representation for inference. The VerbNet classes were associated with event frames within an *Event Structure Lexicon (ESL)* (Im and Pustejovsky, 2010), encoding the subevent structure of the predicate. Consider the verb classes change_of_location and change_of_possession. For instance, the verb *drive* as a change_of_location verb generates the closed domain esl entry shown below.

(3)    drive in *John drove to Boston*
            se1: pre-state: not_be_in (x,y)
             se2: process: driving (x)
             se3: post-state: be_in (x,y)

The goal of this subevent-based VerbNet representation was to facilitate inference and textual entailment tasks. Similarly, Table 1 shows the esl of the verb *arrive*, compared with the semantic frame of the verb in classic VerbNet.

**Table 1:** *arrive* in ESL vs. VerbNet.

**ESL**		**VerbNet**	
**VERB**	**ARRIVE**	**VERB**	**ARRIVE**
CLASS	change_of_location	CLASS	escape-51.1-2
SUB_CLASS	to_goal		
EVENT_TYPE	transition		
SUBEVENT	se1: pre-state: not_be_in (x,y)	SEMANTICS	
	se2: process: arriving(x,y)		motion(during(E))
	se3: post-state: be_in (x,y)		location(end(E),
			theme, oblique)
TEMP_ORDER	se2 ENDS se1		
	se3 MEETS se2		
SENTENCE	*John arrived in Boston*.	EXAMPLE	*He arrived in U.S*.

The above discussion has focused on the identification and encoding of subevent structure for predicative expressions in language. However, in subsequent work within GL (Pustejovsky and Moszkowicz, 2011; Mani and Pustejovsky, 2012), event structure has been enriched to not only *encode* but dynamically *track* those object attributes modified in the course of the event (the location of the moving entity, the extent of a created or destroyed entity, etc.) The resulting event structure representation is called a *Dynamic Event Structure* (Pustejovsky, 2013). Starting with the view that subevents of a complex event can be modeled as a sequence of states (containing formulae), a dynamic event structure explicitly labels the transitions that move an event from state to state (i.e., programs).

A dynamic approach to modeling updates makes a distinction between formulae, ϕ, and programs, π (Harel et al., 2000). A formula is interpreted as a classical propositional expression, with assignment of a truth value in a specific state in the model: e.g., “The glass is broken” is interpreted as a proposition, *broken*(*g*), that is true in a state, *s*, in the model. For our purposes, a state is a set of propositions with assignments to individual variables at a specific frame. We can think of atomic programs as input/output relations, i.e., relations from states to states, and hence interpreted over an input/output state-state pairing: e.g., “The glass broke” is interpreted as a program, α:*s*_1_ → *s*_2_, where at state *s*_1_, the proposition, ¬*broken*(*x*), is true, and at *s*_2_, the proposition, *broken*(*x*) is true. The model encodes three kinds of representations: (i) predicative **content** of a frame; (ii) **programs** that move from frame to frame; and **tests** that must be satisfied for a program to apply. These include: pre-tests, while-tests, and result-tests.

### 2.3. GL-VerbNet

A further step toward a proper subeventual meaning representation is proposed in Brown et al. (2018, 2019), where it is argued that, in order to adequately model change, the VerbNet representation must track the change in the assignment of values to attributes as the event unfolds. This includes making explicit any *predicative opposition* denoted by the verb. For example, simple transitions (achievements) encode either an intrinsic predicate opposition (*die* encodes going from ¬*dead*(*e*_1_, *x*) to *dead*(*e*_2_, *x*)), or a specified relational opposition (*arrive* encodes going from ¬*loc*_*at*(*e*_1_, *x, y*) to *loc*_*at*(*e*_2_, *x, y*)). Creation predicates and accomplishments generally also encode predicate oppositions. As we will describe briefly, GL's event structure and its temporal sequencing of subevents solves this problem transparently, while maintaining consistency with the idea that the sentence describes a single matrix event, *E*.

With the introduction of GL's event structure, the biggest change to VerbNet is the move from a tripartite division of the temporal span of any event [i.e., the division into start(E), during(E), and end(E)] to a model with explicitly quantified (and indexed) subevents, which can be increased or decreased to accommodate the complexity of the event and are ordered using Allen's relational calculus (Allen, 1983). This also eliminates the need for the second-order logic of **start**(E), **during**(E), and **end**(E), allowing for more nuanced temporal relationships between subevents. The default assumption in this new schema is that *e*_1_ precedes *e*_2_, which precedes *e*_3_, and so on. When appropriate, however, more specific predicates can be used to specify other relationships, such as **meets**(*e*_2_, *e*_3_) to show that the end of *e*_2_ meets the beginning of *e*_3_, or **co-temporal**(*e*_2_, *e*_3_) to show that *e*_2_ and *e*_3_ occur simultaneously. The latter can be seen in Section 3.1.4 with the example of accompanied motion.

The second significant change is how causation is represented. In Classic VerbNet, the semantic form implied that the entire atomic event is caused by an Agent, i.e., **cause**(Agent, E), as seen in 4.

(4)     Classic Verbnet representation
            *The lion tamer jumped the lions through the hoop*.
            **motion**(during(E), Theme)
            **path_rel**(start(E), Theme, ?Initial_location, ch_of_loc, prep)
            **path_rel**(during(E), Theme, Trajectory, ch_of_loc, prep)
            **path_rel**(end(E), Theme, ?Destination, ch_of_loc, prep)
            **cause**(Agent, E)

In contrast, in revised GL-VerbNet, “events cause events.” Thus, something an agent does [e.g., **do**(*e*_2_, Agent)] causes a state change or another event [e.g., **motion**(*e*_3_, Theme)], which would be indicated with **cause**(*e*_2_, *e*_3_). This is seen in (5) below, where $\ddot{e_{i}}$ denotes a process event variable.

(5)    GL-Verbnet representation
            *The lion tamer jumped the lions through the hoop*.
            Agent V Theme {PREP} Destination
            **has_location**(*e*_1_, Theme, ?Initial_Location)
            **do**(*e*_2_, Agent)
            **motion**(ë_3_, Theme, Trajectory)
            **¬has_location**(*e*_4_, Theme, ?Initial_location)
            **has_location**(*e*_4_, Theme, ?Destination)
            **cause**(*e*_2_, *e*_3_)

As with Classic VerbNet, each GL-VerbNet class is still defined by a set of members, thematic roles for the predicate-argument structure of these members, selectional restrictions on the arguments, and frames consisting of a syntactic description and a corresponding semantic representation. The semantic representations in each GL-VerbNet class are also still compatible with the member verbs and the syntactic frames of the class. This pairing of each syntactic frame in a class with a semantic representation is a unique feature of VerbNet that emphasizes the close interplay of syntax and semantics. The semantic information is still expressed as a conjunction of semantic predicates, such as **has_state, emit**, or **cause**, and an event variable, either **E** or **e** (see Section 3.2.3 for an explanation of the event variable types). This conjunction of predicates more closely tracks the participants through the various stages of the event evoked by the syntactic frame. For example, an intransitive frame in the class Run-51.3.2 is shown in 6, with the final 4 lines making up the semantic representation, as a conjunction of predicates.

(6)    GL-Verbnet representation
            *The horse ran into the barn*.
            NP V PP
            Theme V Destination
            **has_location**(*e*_1_, Theme, ?Initial_Location)
            **motion**(ë_3_, Theme, ?Trajectory)
            **¬has_location**(*e*_2_, Theme, ?Initial_Location)
            **has_location**(*e*_4_, Theme, Destination)

The arguments of each predicate are represented using the thematic roles for the class. These roles provide the link between the syntax and the semantic representation. Each participant mentioned in the syntax, as well as necessary but unmentioned participants, are accounted for in the semantics. For example, the second component of the first **has_location** semantic predicate above includes an unidentified Initial_Location. That role is expressed overtly in other syntactic alternations in the class (e.g., *The horse ran from the barn*), but in this frame its absence is indicated with a question mark in front of the role. Temporal sequencing is indicated with subevent numbering on the event variable *e*.

A class's semantic representations capture generalizations about the semantic behavior of the member verbs *as a group*. For some classes, such as the Put-9.1 class, the verbs are semantically quite coherent (e.g., *put, place, situate*) and the semantic representation is correspondingly precise 7.

(7)    GL-Verbnet representation
            *I put the book on the table*.
            NP V NP PP
            Agent V Theme {PREP} Destination
            **has_location**(*e*_1_, Theme, ?Initial_Location)
            **do**(*e*_2_, Agent)
            **motion**(ë_3_, Theme, ?Trajectory)
            **¬has_location**(*e*_4_, Theme, ?Initial_location)
            **has_location**(*e*_4_, Theme, Destination)
            **cause**(*e*_2_, *e*_3_)

Other classes, such as Other Change of State-45.4, contain widely diverse member verbs (e.g., *dry, gentrify, renew, whiten*). The representation must be very general to apply to all the verbs. The following representation captures the change of the Patient from its initial state to its final state but ignores the specific type of state change in the example sentence (i.e., from frozen to thawed) in order to be general enough for any verb in the class when used in a basic transitive sentence.

(8)    GL-Verbnet representation
            *Nicholas thawed the meat*.
            NP V NP
            Agent V Patient
            **¬has_state**(*e*_1_, Patient, V_Final_State)
            **do**(*e*_2_, Agent)
            **has_state**(*e*_3_, Patient, V_Final_State)
            **cause**(*e*_2_, *e*_3_)

Classic VerbNet semantic representations were similar to the new ones in several ways: a class's thematic roles were used as arguments to the semantic predicates, and subevents were situated in a temporal ordering. However, as described in Section 2.1, the representation only allowed for three temporal phases: **Start**, **During**, and **End**, which limited the granularity of the subevents and their sequencing. No predicates existed to show causal relationships between the subevents, and semantically necessary roles that were never explicit in the syntax of a class could not be referred to. For example, in the Instrument_Communication-37.4.1 class, no mention could be made of the instrument that was incorporated in every verb in the class (e.g., *phone, cable, radio*).

(9)    Classic Verbnet representation
            *Heather emailed the news to Sarah*.
            Agent V Topic Recipient
            **transfer_info** (during(E), Agent, Recipient, Topic)
            **cause**(Agent, E)

To achieve the expressiveness we wanted in the revised semantic representations, we identified several desiderata:

- Participants clearly tracked across an event for changes in location, existence or other states.
- Flexibility in the number of subevents.
- Subevents related within a representation for causality, temporal sequence and, where appropriate, aspect.
- Predicates consistently used across classes and hierarchically related for flexible granularity.
- Verb-specific features incorporated in the semantic representations where possible.
- Roles to refer to semantically relevant but syntactically absent participants.

Incorporating all these changes consistently across 5,300 verbs posed an enormous challenge, requiring a thoughtful methodology, as discussed in the following section.

### 2.4. Method

VerbNet currently has 329 different classes of verbs and over 1,600 semantic representations. A major revision of these required several stages of development. To begin, we identified consistencies in the types of events across VerbNet classes and then grouped all classes by a short list of basic event types:

- Change of location.
- Change of state.
- Change of possession.
- Transfer of information.
- States.
- Processes.
- Other.

By far the most common event types were the first four, all of which involved some sort of change to one or more participants in the event. We developed a basic first-order-logic representation that was consistent with the GL theory of subevent structure and that could be adapted for the various types of change events. We preserved existing semantic predicates where possible, but more fully defined them and their arguments and applied them consistently across classes. In this first stage, we decided on our system of subevent sequencing and developed new predicates to relate them. We also defined our event variable e and the variations that expressed aspect and temporal sequencing. At this point, we only worked with the most prototypical examples of changes of location, state and possession and that involved a minimum of participants, usually Agents, Patients, and Themes.

Once our fundamental structure was established, we adapted these basic representations to events that included more event participants, such as Instruments and Beneficiaries. We applied them to all frames in the Change of Location, Change of State, Change of Possession, and Transfer of Information classes, a process that required iterative refinements to our representations as we encountered more complex events and unexpected variations.

The next stage involved developing representations for classes that primarily dealt with states and processes. Because our representations for change events necessarily included state subevents and often included process subevents, we had already developed principles for how to represent states and processes.

The final category of classes, “Other,” included a wide variety of events that had not appeared to fit neatly into our categories, such as perception events, certain complex social interactions, and explicit expressions of aspect. However, we did find commonalities in smaller groups of these classes and could develop representations consistent with the structure we had established. Many of these classes had used unique predicates that applied to only one class. We attempted to replace these with combinations of predicates we had developed for other classes or to reuse these predicates in related classes we found.

## 3. Results

In revising these semantic representations, we made changes that touched on every part of VerbNet. Within the representations, we adjusted the subevent structures, number of predicates within a frame, and structuring and identity of predicates. Changes to the semantic representations also cascaded upwards, leading to adjustments in the subclass structuring and the selection of primary thematic roles within a class. In this section we will go through the details of these changes. To give an idea of the scope, as compared to VerbNet version 3.3.2, only seven out of 329—just 2%—of the classes have been left unchanged. We have added 3 new classes and subsumed two others into existing classes. Within existing classes, we have added 25 new subclasses and removed or reorganized 20 others. 88 classes have had their primary class roles adjusted, and 303 classes have undergone changes to their subevent structure or predicates. Our predicate inventory now includes 162 predicates, having removed 38, added 47 more, and made minor name adjustments to 21. All of the rest have been streamlined for definition and argument structure.

### 3.1. Application of GL to VerbNet Representations

Using the Generative Lexicon subevent structure to revise the existing VerbNet semantic representations resulted in several new standards in the representations' form. As discussed in Section 2.2, applying the GL Dynamic Event Model to VerbNet temporal sequencing allowed us refine the event sequences by expanding the previous three-way division of **start(E)**, **during(E)**, and **end(E)** into a greater number of subevents if needed. These numbered subevents allow very precise tracking of participants across time and a nuanced representation of causation and action sequencing within a single event. In the general case, *e*_1_ occurs before *e*_2_, which occurs before *e*_3_, and so on. We've further expanded the expressiveness of the temporal structure by introducing predicates that indicate temporal and causal relations between the subevents, such as **cause**(*e*_*i*_, *e*_*j*_) and **co-temporal**(*e*_*i*_, *e*_*j*_).

Second, we followed GL's principle of using states, processes and transitions, in various combinations, to represent different Aktionsarten. We use E to represent states that hold throughout an event and ë_*n*_ to represent processes. Transitions are *e*_*n*_, as are states that hold for only part of a complex event. These can usually be distinguished by the type of predicate-either a predicate that brings about change, such as **transfer**, or a state predicate like **has_location**. Our representations of accomplishments and achievements use these components to follow changes to the attributes of participants across discrete phases of the event.

Finally, the Dynamic Event Model's emphasis on the opposition inherent in events of change inspired our choice to include pre- and post-conditions of a change in all of the representations of events involving change. Previously in VerbNet, an event like “eat” would often begin the representation at the **during(E)** phase. This type of structure made it impossible to be explicit about the opposition between an entity's initial state and its final state. It also made the job of tracking participants across subevents much more difficult for NLP applications. Understanding that the statement 'John dried the clothes' entailed that the clothes began in a wet state would require that systems infer the initial state of the clothes from our representation. By including that initial state in the representation explicitly, we eliminate the need for real-world knowledge or inference, an NLU task that is notoriously difficult.

One way we captured this opposition was by adding this type of initial state predicate in those representations of change that did not already have one. We also greatly expanded the use of negated predicates to make explicit the opposition: e.g., *John died* is analyzed as the opposition 〈**alive**,(*e*_1_,Patient), ¬**alive**,(*e*_2_,Patient)〉. In (10), we use the opposition between **has_location** and **¬has_location** to make clear that once the Theme is in motion (in *e*_2_), it is no longer at the Initial_location.

(10)    GL-Verbnet representation
              *The rabbit hopped across the lawn*.
              Theme V Trajectory
              **has_location**(*e*_1_, Theme, ?Initial_Location)
              **motion**(ë_2_, Theme, Trajectory)
              **¬has_location**(*e*_2_, Theme, ?Initial_location)
              **has_location**(*e*_3_, Theme, ?Destination)

Although people infer that an entity is no longer at its initial location once motion has begun, computers need explicit mention of this fact to accurately track the location of the entity (see Section 3.1.3 for more examples of opposition and participant tracking in events of change).

#### 3.1.1. States

Like the classic VerbNet representations, we use *E* to indicate a state that holds throughout an event. For this reason, many of the representations for state verbs needed no revision, including the representation from the Long-32.2 class.

(11)    Classic and GL-Verbnet representation
              *Danny longed for a sunny day*.
              **desire**(E, Pivot, Theme)

Sixteen classes, however, used the **During(E)** format to represent states, such as Exist-47.1, Intend-61.2, and Contain-15.4. The last, for example, was changed from **contain**(During(E), Pivot, Theme) to **contain**(E, Pivot, Theme). In other cases, the change was in the opposite direction, from state to process. The representation in the Exhale-40.1.3 class included **body_process**(E, Agent), which has been changed to **body_process**(ë_*n*_, Agent).

#### 3.1.2. Processes

Process subevents were not distinguished from other types of subevents in previous versions of VerbNet. They often occurred in the **During(E)** phase of the representation, but that phase was not restricted to processes. With the introduction of ë, we can not only identify simple process frames but also distinguish punctual transitions from one state to another from transitions across a longer span of time; that is, we can distinguish accomplishments from achievements.

Eighteen classes in GL-VerbNet group together simple process verbs. Examples include Snooze-40.4, with the predicate **sleep**(ë, Agent); Simple_dressing-41.3.1-1, with the predicate **wear**(ë, Agent); and Work-73.2, with the predicates **work**(ë, Agent, Theme) and **cooperate**(ë, Agent, Co-Agent, Theme). Of course, adding temporal or locational information to a sentence expressing a process can transform it into an achievement (e.g., “He slept *for an hour*.” or “He ran *home*.”) VerbNet only includes such phrases in its syntactic frames when they are obligatory (e.g., “She put the book *on the table*”) or when they are part of key syntactic alternations that distinguish a class, such as the dative alternation in the Give-13.1 class.

Processes are very frequently subevents in more complex representations in GL-VerbNet, as we shall see in the next section. For example, representations pertaining to changes of location usually have **motion**(ë, Agent, Trajectory) as a subevent.

#### 3.1.3. Change Events

Many of the changes inspired by the Generative Lexicon's Dynamic Event Model come together in the 163 VerbNet classes that focus on events of change. These range from change of location classes like Run-51.3.2 (39 classes); to change of possession classes like Give-13.1 (18 classes); to information transfer classes like Lecture-37.11 (25 classes); to changes of state like Become-109.1 (81 classes). We give examples here of the prototypical form used for these events, along with some variations. Further examples can be found in Brown et al. (2018, 2019). Some refinements have since been made to the representations, so this article should be considered the authority for any differences that can be found.

The Escape-51.1 class is a typical change of location class, with member verbs like *depart, arrive* and *flee*. The most basic change of location semantic representation (12) begins with a state predicate **has_location**, with a subevent argument *e*_1_, a Theme argument for the object in motion, and an Initial_location argument. The **motion** predicate (subevent argument *e*_2_) is underspecified as to the manner of motion in order to be applicable to all 40 verbs in the class, although it always indicates translocative motion. Subevent *e*_2_ also includes a negated **has_location** predicate to clarify that the Theme's translocation away from the Initial Location is underway. A final **has_location** predicate indicates the Destination of the Theme at the end of the event. As mentioned earlier, not all of the thematic roles included in the representation are necessarily instantiated in the sentence.

(12)    GL-Verbnet representation
              *Natasha came to Colorado*.
              **has_location**(*e*_1_, Theme, ?Initial_Location)
              **motion**(ë_2_, Theme, ?Trajectory)
              **¬has_location**(*e*_2_, Theme, ?Initial_location)
              **has_location**(*e*_3_, Theme, Destination)

This representation follows the GL model by breaking down the transition into a process and several states that trace the phases of the event.

Representations for changes of state take a couple of different, but related, forms. For those state changes that we construe as punctual or for which the verb does not provide a syntactic slot for an Agent or Causer, we use a basic opposition between state predicates, as in the Die-42.4 and Become-109.1 classes. 13 shows a simple version of this format.

(13)    GL-Verbnet representation
              *The belt came undone*.
              **¬has_state**(*e*_1_, Theme, Result)
              **has_state**(*e*_2_, Theme, Result)

State changes with a notable transition or cause take the form we used for changes in location, with multiple temporal phases in the event. The similarity can be seen in 14 from the Tape-22.4 class, as can the predicate we use for Instrument roles.

(14)    GL-Verbnet representation
              *Linda stitched the front to the back with green thread*.
              **¬attached**(*e*_1_, Patient, Co-patient)
              **do**(*e*_2_, Agent)
              **utilize**(*e*_2_, Agent, Instrument)
              **attached**(*e*_3_, Patient, Co-patient)
              **cause**(*e*_2_, *e*_3_)

A final pair of examples of change events illustrates the more subtle entailments we can specify using the new subevent numbering and the variations on the event variable. Changes of possession and transfers of information have very similar representations, with important differences in which entities have possession of the object or information, respectively, at the end of the event. In 15, the opposition between the Agent's possession in *e*_1_ and non-possession in *e*_3_ of the Theme makes clear that once the Agent transfers the Theme, the Agent no longer possesses it. However, in 16, the E variable in the initial **has_information** predicate shows that the Agent retains knowledge of the Topic even after it is transferred to the Recipient in *e*_2_.

(15)    GL-Verbnet representation
              *I gave my dog a treat*.
              **has_possession**(*e*_1_, Agent, Theme)
              **¬has_possession**(*e*_1_, Recipient, Theme)
              **transfer**(*e*_2_, Agent, Theme, Recipient)
              **has_possession**(*e*_3_, Recipient, Theme)
              **¬has_possession**(*e*_3_, Agent, Theme)
              **cause**(*e*_2_, *e*_3_)

(16)    GL-Verbnet representation
              *Carlos told Stella about his vacation*.
              **has_information**(E, Agent, Topic)
              **transfer_info**(*e*_1_, Agent, Topic, Recipient)
              **has_information**(*e*_2_, Recipient, Topic)
              **cause**(*e*_1_, *e*_2_)

#### 3.1.4. Subevent-Subevent Relations

We have introduced or revised eight predicates that refine the temporal and causal relations between subevents: **start**, **finish**, **meets**, **overlaps**, **co-temporal**, **repeated_sequence**, **cause**, and **in_reaction_to**. In the semantic representations, the subevent sequencing indicated by the numbered subevent variables is enough to express the time sequence when subevents follow a simple progression. However, subevents can occur simultaneously or overlap. Predicates that take two or more subevent variables as arguments clarify what would otherwise be misleading subevent numbering. For example, in (17), **start**(*e*_2_, *e*_3_) clarifies the relationship between the party (*e*_3_) and the speech (*e*_2_). This predicate has been defined as “One subevent is the beginning portion of another subevent.”

(17)    GL-Verbnet representation
              *I began the party with a speech*.
              Agent V Eventuality with Subeventuality
              **¬occur**(*e*_1_, Eventuality)
              **engage_in**(*e*_2_, Agent, Subeventuality)
              **occur**(ë_3_, Eventuality)
              **start**(*e*_2_, ë_3_)

### 3.2. Predicate Structure and Types

Another significant change to the semantic representations in GL-VerbNet was overhauling the predicates themselves, including their definitions and argument slots. We added 47 new predicates, two new predicate types, and improved the distribution and consistency of predicates across classes. Improving the preexisting predicates involved streamlining their internal structure, providing each with a clear definition and clear descriptions of the roles played by each argument slot, and creating hierarchies through which the predicates are linked to one another according to shared semantics, aspectual behavior, and valency patterns. Within the representations, new predicate types add much-needed flexibility in depicting relationships between subevents and thematic roles. As we worked toward a better and more consistent distribution of predicates across classes, we found that new predicate additions increased the potential for expressiveness and connectivity between classes. We also replaced many predicates that had only been used in a single class. In this section, we demonstrate how the new predicates are structured and how they combine into a better, more nuanced, and more useful resource. For a complete list of predicates, their arguments, and their definitions (see Appendix A).

#### 3.2.1. Change in Predicate Structure

Introducing consistency in the predicate structure was a major goal in this aspect of the revisions. In Classic VerbNet, the basic predicate structure consisted of a time stamp (**Start**, **During**, or **End** of E) and an often inconsistent number of semantic roles. The time stamp pointed to the phase of the overall representation during which the predicate held, and the semantic roles were taken from a list that included thematic roles used across VerbNet as well as constants, which refined the meaning conveyed by the predicate. Some predicates could appear with or without a time stamp, and the order of semantic roles was not fixed. For example, the Battle-36.4 class included the predicate **manner**(manner, Agent), where a constant that describes the manner of the Agent fills in for manner. While **manner** did not appear with a time stamp in this class, it did in others, such as Bully-59.5 where it was given as **manner**(E, manner, Agent). In some classes, the order of its two semantic arguments was reversed.

Each argument slot within a given predicate was restricted to a limited set of thematic roles. In the Exchange-13.6.1 class, the **path_rel** predicate included two argument slots for thematic roles: one for the possessor of a thing during the phase, and the other for the thing itself. Like **manner**, the order of these two arguments was not always consistent. **Path_rel** was used for all kinds of change events, including change of location, change of state, and transfer of possession; when it was used in transfer of possession classes like Exchange, the possessor argument was limited to thematic roles Source and Goal. Since those roles were not included as primary roles for the class, the representation included additional **equals** predicates equating the class participant named in the syntactic representation and the Source or Goal named in the **path_rel** predicate.

(18)    Classic Verbnet representation
              *Twenty couples exchanged rings*.
              Agent 〈+plural〉 V Theme 〈+plural〉
              **path_rel**(Start(E), Source_I, Theme_I, ch_of_poss, prep)
              **path_rel**(Start(E), Source_J, Theme_J, ch_of_poss, prep)
              **transfer**(During(E), Theme_I)
              **transfer**(During(E), Theme_J)
              **path_rel**(End(E), Goal_J, Theme_I, ch_of_poss, prep)
              **path_rel**(End(E), Goal_I, Theme_J, ch_of_poss, prep)
              **cause**(Agent, E)
              **equals**(Source_I, Agent)
              **equals**(Goal_J, Agent)
              **equals**(Source_J, Agent)
              **equals**(Goal_I, Agent)
              **opposition**(Initial_State, Result)

Having an unfixed argument order was not usually a problem for the **path_rel** predicate because of the limitation that one argument must be of a Source or Goal type. But in some cases where argument order was not applied consistently and an Agent role was used, it became difficult for both humans and computers to track whether the Agent was initiating the overall event or just the particular subevent containing the predicate.

The new predicates eliminate the inconsistencies and ambiguities of the old and define three separate predicate types. Each predicate now comes with a clear definition and a fixed set of argument slots, each with a clear description of what the argument named in the slot contributes to the overall situation or relation the predicate describes. That the argument slots are fixed indicates that each argument is an essential part of the situation. If a predicate is used in a class for which certain slots are never realized as explicit verb arguments, that predicate argument should still be understood to be an integral part of the semantic representation, and the slot will be filled by an essential role (Palmer, 1990) or a constant. Note the new representation for the same sentence in the Exchange class:

(19)    GL-Verbnet representation
              *Twenty couples exchanged rings*.
              Agent 〈+plural〉 V Theme 〈+plural〉
              **has_possession**(*e*_1_, Agent_I, Theme_I)
              **¬has_possession**(*e*_1_, Agent_J, Theme_I)
              **has_possession**(*e*_2_, Agent_J, Theme_J)
              **¬has_possession**(*e*_2_, Agent_I, Theme_J)
              **transfer**(*e*_3_, Agent_I, Theme_I, Agent_J)
              **transfer**(*e*_4_, Agent_J, Theme_J, Agent_I)
              **has_possession**(*e*_5_, Agent_J, Theme_I)
              **¬has_possession**(*e*_5_, Agent_I, Theme_I)
              **has_possession**(*e*_6_, Agent_I, Theme_J)
              **¬has_possession**(*e*_6_, Agent_J, Theme_J)
              **cause**(*e*_3_, *e*_5_)
              **cause**(*e*_4_, *e*_6_)

The first major change to this representation was that **path_rel** was replaced by a series of more specific predicates depending on what kind of change was underway. Here, it was replaced by **has_possession**, which is now defined as “A participant has possession of or control over a Theme or Asset.” It has three fixed argument slots of which the first is a time stamp, the second is the possessing entity, and the third is the possessed entity. These slots are invariable across classes and the two participant arguments are now able to take any thematic role that appears in the syntactic representation or is implicitly understood, which makes the **equals** predicate redundant. It is now much easier to track the progress of a single entity across subevents and to understand who is initiating change in a change predicate, especially in cases where the entity called Agent is not listed first.

#### 3.2.2. Basic Predicate Types

The new predicates are divided into three basic structural types: situation predicates, relation predicates, and subevent modifier predicates. Situation predicates make up the majority of the predicate inventory and describe events and states; each has a time stamp as its first argument slot and thematic roles or constants in the remaining slots. A typical situation predicate is **has_location**(*e*_4_, Theme, Destination). Every semantic representation and every subevent must include at least one situation predicate.

Relation predicates come in two varieties: subevent-subevent relations, and role-role relations. As per Section 3.1.4, subevent-subevent relations take two or more subevent variables as their arguments and describe a causal or temporal relation that holds between them, such as **cause**(*e*_3_, *e*_5_). Role-role relations describe relationships between thematic roles, for example **part_of**(Theme, Agent), which says that the Theme is a constituent part of the Agent.

Sometimes a thematic role in a class refers to an argument of the verb that is an eventuality. Because it is sometimes important to describe relationships between eventualities that are given as subevents and those that are given as thematic roles, we introduce as our third type subevent modifier predicates, for example, **in_reaction_to**(*e*_1_, Stimulus). Here, as well as in subevent-subevent relation predicates, the subevent variable in the first argument slot is not a time stamp; rather, it is one of the related parties. **In_reaction_to**(*e*_1_, Stimulus) should be understood to mean that subevent *e*_1_ occurs as a response to a Stimulus. Subevent modifier predicates also include monovalent predicates such as **irrealis**(*e*_1_), which conveys that the subevent described through other predicates with the *e*_1_ time stamp may or may not be realized.

#### 3.2.3. Subevent Variable Types

Section 3.1 introduced the new subevent variables *e*_*n*_, E, and ë. Here, we showcase the finer points of how these different forms are applied across classes to convey aspectual nuance. As we saw in example 11, E is applied to states that hold throughout the run time of the overall event described by a frame. When E is used, the representation says nothing about the state having beginning or end boundaries other than that they are not within the scope of the representation. This is true whether the representation has one or multiple subevent phases.

Similarly, ë is in and of itself associated with ongoing processes, i.e., the process event variable in Pustejovsky (1995, 2013). It is not quite parallel to E; there are no instances of ë that describe a process that holds throughout an entire representation while other subevents go on in sub-phases within it. Rather, ë can be used to head one of multiple subevents (as in the **motion** example 10) or it can be the only subevent, as in (20) below from Snooze-40.4:

(20)    GL-Verbnet representation
              *Gloria snoozed*.
              Agent V
              **body_process**(ë, Agent)
              **sleep**(ë, Agent)

In multi-subevent representations, ë conveys that the subevent it heads is unambiguously a process for all verbs in the class. If some verbs in a class realize a particular phase as a process and others do not, we generalize away from ë and use the underspecified e instead. A process described by ë is ongoing throughout its entire subevent. If a representation needs to show that a process begins or ends during the scope of the event, it does so by way of pre- or post-state subevents bookending the process. The exception to this occurs in cases like the Spend_time-104 class (21) where there is only one subevent. The verb describes a process but bounds it by taking a Duration phrase as a core argument. For this, we use a single subevent *e*_1_ with a subevent-modifying **duration** predicate to differentiate the representation from ones like (20) in which a single subevent process is unbounded.

(21)    GL-Verbnet representation
              *She spent five years waiting tables*.
              Agent V Duration Eventuality
              **spend_time**(*e*_1_, Agent, Eventuality)
              **duration**(*e*_1_, Duration)

Another pair of classes shows how two identical state or process predicates may be placed in sequence to show that the state or process continues past a could-have-been boundary. In example 22 from the Continue-55.3 class, the representation is divided into two phases, each containing the same process predicate. This predicate uses ë because, while the event is divided into two conceptually relevant phases, there is no functional bound between them.

(22)    GL-Verbnet representation
              *He continued to pack*.
              Agent V Eventuality
              **engage_in**(ë_1_, Agent, Eventuality)
              **engage_in**(ë_2_, Agent, Eventuality)

As we will see in Section 3.3.2, each situation predicate is associated with its own typical Aktionsart which informs the type of e it takes as a time stamp. However, since subevent variables often pick out a period of time during which multiple predicates hold, a decision must be made as to which type of e best characterizes the set. Since E can only be used for states that hold through an entire representation, all predicates headed with it must meet this criterion. States that hold while a transition takes place (especially, stative predicates like **contact**) inherit the event variable type of the transition, ë or e.

#### 3.2.4. Verb-Specific and Predicate-Specific Roles

Because we have enabled predicates to select thematic roles that are not realized as verb arguments in the syntactic representation, we introduce two new role types that appear as predicate arguments: predicate-specific and verb-specific. Predicate-specific roles are semantically essential arguments projected by the predicates. In the event that a VerbNet class does not provide a primary thematic role for such an argument, we give the argument a special “PredSpecific” type so that it remains easily distinguishable from primary thematic roles. These roles are critical to tracking the behavior of participants through the subevents. The Trajectory or path argument of **motion** predicates is frequently realized as predicate-specific, since many change of location classes do not consider it to be a primary argument of their verbs. Clearly, it is still essential to understanding a translocation event, especially for spatial processing systems that tie language to real-world motion events. In 23 and 24, both the Initial_Location and Trajectory roles are predicate specific.

Sometimes critical entities or attributes involved in an event are encoded within the verb itself. VerbNet includes many classes that are organized specifically according to this behavior, for example the Tape-22.4 class, in which the verb names the Instrument of affixing; the Pocket-9.10 class, in which the verb names the Destination; and the Amuse-31.1 class, in which the verb names an Emotion. Before now, there was no way to incorporate this critical information into the semantic representations. Because some classes differ from one another according to this behavior alone, we were losing inter-class specificity as well. All verb-specific roles begin with the prefix “V_” and end with either a typical thematic role or a constant, making them easily distinguishable from any instances of a syntactically explicit realization of the same argument. In the examples from Pocket-9.10 below, note the change from V_Destination to Destination when an explicit Destination is added to the sentence:

(23)    GL-Verbnet representation
              *Lydia pocketed the change*.
              **has_location**(*e*_1_, Theme, Initial_Location)
              **do**(*e*_2_, Agent)
              **motion**(ë_3_, Theme, Trajectory)
              **¬has_location**(ë_3_, Theme, Initial_Location)
              **has_location**(*e*_4_, Theme, V_Destination)
              **cause**(*e*_2_, ë_3_)

(24)    GL-Verbnet representation
              *We bottled the cider in one-gallon jugs*.
              **has_location**(*e*_1_, Theme, Initial_Location)
              **do**(*e*_2_, Agent)
              **motion**(ë_3_, Theme, Trajectory)
              **¬has_location**(ë_3_, Theme, Initial_Location)
              **has_location**(*e*_4_, Theme, Destination)
              **cause**(*e*_1_, ë_3_)

### 3.3. Predicate Coherence

In addition to substantially revising the representation of subevents, we increased the informativeness of the semantic predicates themselves and improved their consistency across classes. This effort included defining each predicate and its arguments and, where possible, relating them hierarchically in order for users to chose the appropriate level of meaning granularity for their needs. We also strove to connect classes that shared semantic aspects by reusing predicates wherever possible. In some cases this meant creating new predicates that expressed these shared meanings, and in others, replacing a single predicate with a combination of more primitive predicates.

#### 3.3.1. Replacing Uninformative or Redundant Predicates

One obvious way to increase semantic coherence was to choose a single predicate to represent opposing predicates and use negation to show the opposition. For example, Classic VerbNet had the predicate **apart** in the Separate-23.1, Split-23.2, and Disassemble-23.3 classes, but the predicate **attached** in the Tape-22.4 class. We eliminated **apart** and used **attached** and **¬attached** throughout these classes. Similarly, we found three semantically similar classes in different areas of VerbNet that each had only a single predicate used in no other classes: The representation in the Confront-98 class *(Lee tackled the problem)* was simply **confront**(During(E), Agent, Theme, Instrument); in Cope-83 *(She handled the unruly customers)* it was **cope**(E, Agent, Theme), and in Neglect-75.1 *(He failed to do the job)*, it was **neglect**(E, Agent, Theme). We replaced all three with either **handle** or **¬handle**.

In thirty classes, we replaced single predicate frames (especially those with predicates found in only one class) with multiple predicate frames that clarified the semantics or traced the event more clearly. For example, (25) and (26) show the replacement of the **base** predicate with more general and more widely-used predicates.

(25)    Classic Verbnet representation
              *We based our plans on his information*.
              **cause**(Agent, E)
              **base**(E, Theme, Source)

(26)    GL-Verbnet representation
              *We based our plans on his information*.
              **support**(E, Source, Theme)
              **engage_in**(*e*_1_, Agent, Theme)
              **utilize**(*e*_1_, Agent, Source)

Using the **support** predicate links this class to deduce-97.2 and support-15.3 *(She supported her argument with facts)*, while **engage_in** and **utilize** are widely used predicates throughout VerbNet.

#### 3.3.2. Predicate Taxonomies by Aktionsart, Semantic Roles and Valency

We have organized the predicate inventory into a series of taxonomies and clusters according to shared aspectual behavior and semantics. These structures allow us to demonstrate external relationships between predicates, such as granularity and valency differences, and in turn, we can now demonstrate inter-class relationships that were previously only implicit.

The first taxonomy (Appendix B) groups predicates into states, processes and perfectives (https://uvi.colorado.edu/references_page). The perfective category includes all events that include a terminus and has been subdivided into achievements and accomplishments. To connect this back to examples we have already seen, **motion** (5) is categorized as a process, **transfer** (15) as a perfective, and **has_location** (5) as a state.

Because predicates must apply evenly across all verbs in a class and because some classes contain an aspectually diverse set of verbs or verbs that may be realized according to more than one Aktionsart within the class, we also include subgroups in this taxonomy for predicates that may be associated with more than one aspectual type (e.g., **emit**, which may represent a process or a perfective). This allows the semantic representations to remain appropriately general while laying a foundation for future work in which they may be expanded in a verb-specific manner.

Although they are not situation predicates, subevent-subevent or subevent-modifying predicates may alter the Aktionsart of a subevent and are thus included at the end of this taxonomy. For example, the **duration** predicate (21) places bounds on a process or state, and the **repeated_sequence**(*e*_1_, *e*_2_, *e*_3_, ...) can be considered to turn a sequence of subevents into a process, as seen in the Chit_chat-37.6, Pelt-17.2, and Talk-37.5 classes. **Irrealis** may be use to make result states optional.

#### 3.3.3. Semantic Clusters

A second, non-hierarchical organization (Appendix C) groups together predicates that relate to the same semantic domain and defines, where applicable, the predicates' relationships to one another. Predicates within a cluster frequently appear in classes together, or they may belong to related classes and exist along a continuum with one another, mirror each other within narrower domains, or exist as inverses of each other. For example, we have three predicates that describe degrees of physical integration with implications for the permanence of the state. **Together** is most general, used for co-located items; **attached** represents adhesion; and **mingled** indicates that the constituent parts of the items are intermixed to the point that they may not become unmixed. **Spend** and **spend_time** mirror one another within sub-domains of money and time, and in fact, this distinction is the critical dividing line between the Consume-66 and Spend_time-104 classes, which contain the same syntactic frames and many of the same verbs. Similar class ramifications hold for inverse predicates like **encourage** and **discourage**.

**Figure 1:**
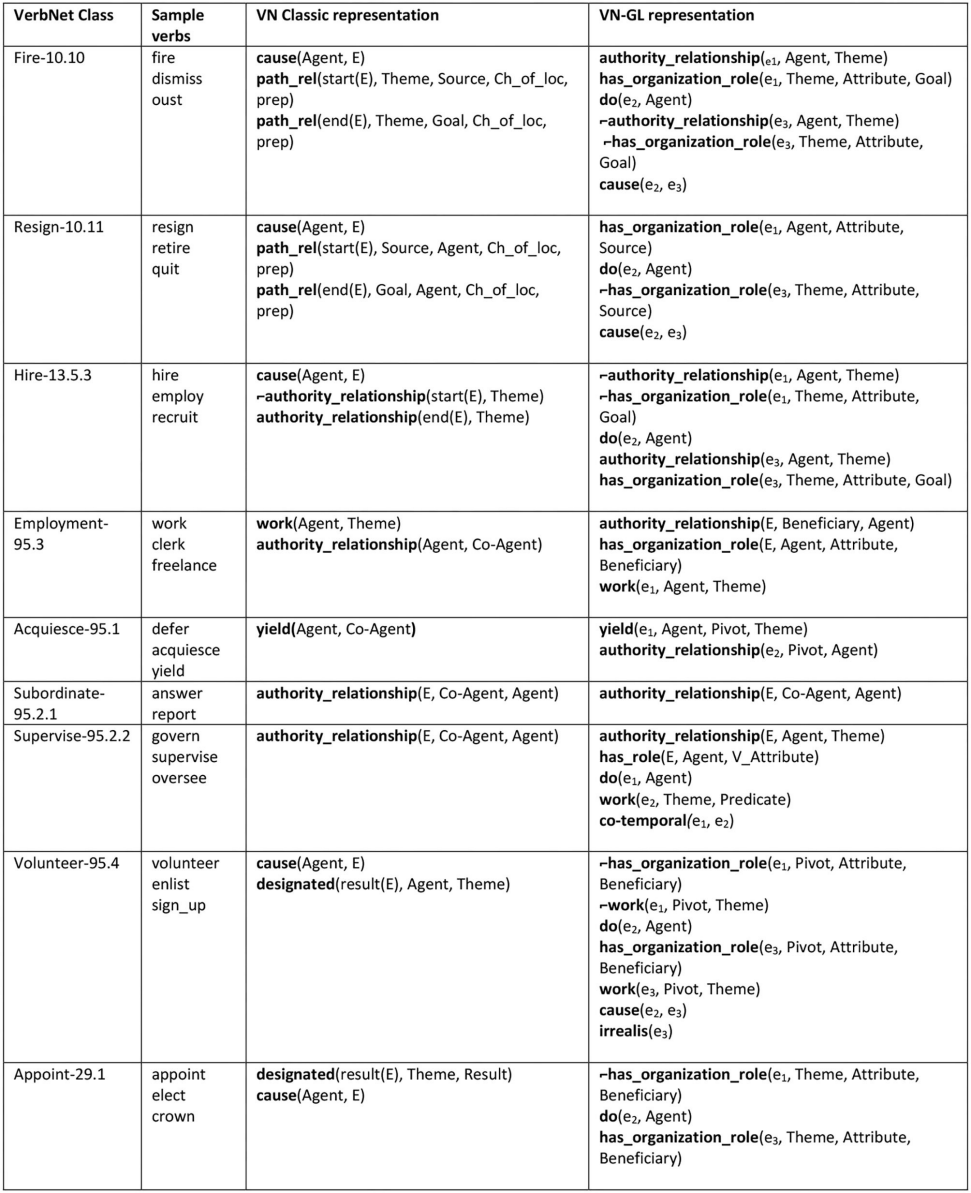
The classes using the organizational role cluster of semantic predicates, showing the Classic VN vs. VN-GL representations.

To get a more comprehensive view of how semantic relatedness and granularity differences between predicates can inform inter-class relationships, consider the organizational-role cluster (Figure 1). This set involves classes that have something to do with employment, roles in an organization, or authority relationships. These classes were spread out across the VerbNet class hierarchy, with some in the removal group (classes suffixed with -10, such as Remove-10.1), the getting group (classes suffixed with -13, such as Give-13.1), the Attribute-related group (suffixed with -29, such as Masquerade-29.6), and an authority-relationship group (suffixed with -95, all included here). The representations for the classes in Figure 1 were quite brief and failed to make explicit some of the employment-related inter-class connections that were implicitly available.

With the aim of improving the semantic specificity of these classes and capturing inter-class connections, we gathered a set of domain-relevant predicates and applied them across the set. **Authority_relationship** shows a stative relationship dynamic between animate participants, while **has_organization_role** shows a stative relationship between an animate participant and an organization. Lastly, **work** allows a task-type role to be incorporated into a representation (*he worked on the Kepler project*).

Fire-10.10 and Resign-10.11 formerly included nothing but two **path_rel**(ch_of_loc) predicates plus **cause**, in keeping with the basic change of location format utilized throughout the other -10 classes. This representation was somewhat misleading, since translocation is really only an occasional side effect of the change that actually takes place, which is the ending of an employment relationship. See Figure 1 for the old and new representations from the Fire-10.10 class.

The new representation for the Hire-13.5.3 class mirrors Fire-10.10 now, clearly demonstrating the connection between these classes. In the past, Hire-13.5.3 consisted solely of an opposition between an **authority_relationship** predicate and its negation, leaving it with no visible connection to Fire-10.10.

Acquiesce-95.1, Supervision-95.2.2, and Subordinate-95.2.1 all consisted of singleton representations before revisions. Acquiesce-95.1 used a single **yield** predicate (a predicate not defined and not found anywhere else in VerbNet—a true singleton) while Supervision-95.2.2 and Subordinate-95.2.1 used just **authority_relationship**. The latter two representations differed only in the ordering of their predicate arguments. To flesh these representations out a bit, we added an **authority_relationship** predicate to Acquiesce-95.1 and expanded not only the representation but the overall scope of Supervision-95.2.2. First, we defined **yield** and contextualized it by placing it in the org-role semantic predicate cluster. With the inclusion of **authority_relationship**, the representation now provides more information about the authority dynamic between the participants within the yielding event and we connect Acquiesce to the rest of the org-role group. Supervision-95.2.2's coverage has expanded considerably with the addition of **work** and **has_role** predicates. We found that all verbs in the class support syntactic frames that incorporate a task-type Theme role (*Martha led the students in a task*), so we added Theme to the class as a primary role along with two new frames exemplifying its use. The class has an additional, previously ignored hallmark: all of the member verbs encode semantics relating to the social role played by the Agent in the event (e.g., *leader*). Since these social roles were not limited to organization-internal positions, we included the coarser-grained **has_role** predicate with a V_Attribute argument pointing back to the verb. This and the other semantic clusters we created demonstrate that having external predicate structures provide a valuable new layer of semantic refinement within and between classes.

## 4. Evaluation

As discussed above, as a broad coverage verb lexicon with detailed syntactic and semantic information, VerbNet has already been used in various NLP tasks, primarily as an aid to semantic role labeling or ensuring broad syntactic coverage for a parser. The richer and more coherent representations described in this article offer opportunities for additional types of downstream applications that focus more on the semantic consequences of an event. The clearer specification of pre- and post-conditions has been useful for automatic story generation (Ammanabrolu et al., 2020; Martin, 2021), while the more consistent incorporation of aspect contributed to a system for automatic aspectual tagging of sentences in context (Chen et al., 2021). However, the clearest demonstration of the coverage and accuracy of the revised semantic representations can be found in the Lexis system (Kazeminejad et al., 2021) described in more detail below.

One of the downstream NLP tasks in which VerbNet semantic representations have been used is tracking entity states at the sentence level (Clark et al., 2018; Kazeminejad et al., 2021). Entity state tracking is a subset of the greater machine reading comprehension task. The goal is to track the changes in states of entities within a paragraph (or larger unit of discourse). This change could be in location, internal state, or physical state of the mentioned entities. For instance, a Question Answering system could benefit from predicting that entity *E* has been destroyed or has moved to a new location at a certain point in the text, so it can update its state tracking model and would make correct inferences. By reading a text describing photosynthesis, for example, it is desirable that a machine will understand in which step and in what location sugar is produced (location and existence state tracking for the entity “sugar,”) even though these state changes are implicit rather than explicitly mentioned. A clear example of that utility of VerbNet semantic representations in uncovering implicit information is in a sentence with a verb such as “carry” (or any verb in the VerbNet *carry-11.4* class for that matter). If we have ◂ *X* *carried* *Y* *to* *Z*▸, we know that by the end of this event, both *Y* and *X* have changed their location state to *Z*. This is not recoverable even if we know that “carry” is a motion event (and therefore has a theme, source, and destination). This is in contrast to a “throw” event where only the theme moves to the destination and the agent remains in the original location. Such semantic nuances have been captured in the new GL-VerbNet semantic representations, and Lexis, the system introduced by Kazeminejad et al., 2021, has harnessed the power of these predicates in its knowledge-based approach to entity state tracking.

Lexis relies first and foremost on the GL-VerbNet semantic representations instantiated with the extracted events and arguments from a given sentence, which are part of the SemParse output (Gung, 2020)—the state-of-the-art VerbNet neural semantic parser. In addition, it relies on the semantic role labels, which are also part of the SemParse output. The state change types Lexis was designed to predict include change of existence (created or destroyed), and change of location. The utility of the subevent structure representations was in the information they provided to facilitate entity state prediction. This information includes the predicate types, the temporal order of the subevents, the polarity of them, as well as the types of thematic roles involved in each.

As an example, for the sentence “The water forms a stream,”^2^, SemParse automatically generated the semantic representation in (27). In this case, SemParse has incorrectly identified the water as the *Agent* rather than the *Material*, but, crucially for our purposes, the Result is correctly identified as the stream. The fact that a Result argument changes from not being (¬**be**) to being (**be**) enables us to infer that at the end of this event, the result argument, i.e., “a stream,” has been created.

(27)    GL-Verbnet representation instantiated by SemParse
              *The water forms a stream*.
              **¬has_state**(*e*_1_, ?_Material, V_Final_State)
              **¬be**(*e*_2_, a stream_Result, V_Final_State)
              **do**(*e*_2_, the water_Agent)
              **has_state**(*e*_3_, ?_Material, V_Final_State)
              **be**(*e*_2_, a stream_Result, V_Final_State)

As mentioned earlier, the new VerbNet semantic representations have been designed to uncover certain kinds of implicit information. Being able to extract such implicit information is critical in downstream NLP tasks such as question answering or machine reading comprehension. The above-mentioned “carry” example has been illustrated in (28). Evidently, the *Theme* (urea and carbon dioxide) and *Agent* (blood) start together at an uninstantiated/unknown Initial Location. A Motion event occurs to both the *Theme* and the *Agent* and as a result, both move away from the *Initial Location*, both move along a *Trajectory*, and, finally, both arrive together at the *Destination* (kidneys). This change of location (moved) for *urea, carbon dioxide*, and *blood* and the resulting ending up in the *kidneys* is exactly what human annotators had indicated in the ProPara dataset (Dalvi et al., 2018). Lexis managed to extract the same information utilizing the VerbNet semantic representation generated by SemParse. Of course, what enables the prediction is a set of heuristic rules which go down a decision tree to predict whether a change of existence or location has happened, and if so, pinpoint the type of change, as well as a potential locus of change, i.e., where did the change occur. An example is occurrence of ¬**be** followed by **be**, pointing to a created state change, or a **be** followed by ¬**be**, pointing to a destroyed state change. There is also a *Destroyed* VerbNet primitive predicate which points to a destroyed state change too. A Motion primitive predicate is among a series of predicates indicating a moved state change. The more challenging part of crafting the rules over the logical predicates is identifying the locus of change: the destination in a moved state change, particularly when the predicate is other than the inherent motion predicates, such as Motion or Transfer; or when the created or destroyed state change has occurred.

(28)    GL-Verbnet representation
              *The blood carries the urea and carbon dioxide to the kidneys*.
              **has_location**(*e*_1_, the blood_Agent, ?Initial_Location)
              **has_location**(*e*_2_,         the         urea         and         carbon
              dioxide_Theme, ?Initial_Location)
              **motion**(ë_3_, the blood_Agent, ?Trajectory)
              **motion**(ë_4_,         the         urea         and         carbon
              dioxide_Theme, ?Trajectory)
              **¬has_location**(*e*_3_, the blood_Agent, Destination)
              **¬has_location**(*e*_4_,    the    urea    and    carbon
              dioxide_Theme, Destination)
              **has_location**(*e*_5_, the blood_Agent, Destination)
              **has_location**(*e*_6_,    the    urea    and    carbon
              dioxide_Theme, Destination)
              **co-temporal**(*e*_3_, *e*_4_)

In addition to VerbNet semantic representations, SemParse returns the PropBank parse for the input sentence. In some cases where the VerbNet parser fails to instantiate an argument (which is mostly due to the relatively small size of available training data), there is still a chance that the PropBank parser succeeds in doing so. For example, in the sentence “The roots absorb water and minerals from the soil,” the VerbNet semantic representation Take In(?_Goal_, water and minerals_Theme_) fails to instantiate the *Goal* argument. However, from the PropBank parse of this sentence we have A0: *The roots*, A1: *water and minerals*, A2: *from the soil*. On the other hand, from the semantics of the primitive predicate Take In, we know that the *Goal* is the same as *A0* (protoAgent). As a result, we can complete the VerbNet semantic representation for the missing value for *Goal* with the value for A0, *The roots*. This is to maximize the symbolic predictive power of Lexis.

We evaluated Lexis on the ProPara dataset (Dalvi et al., 2018). ProPara was designed for the task of entity state tracking on procedural paragraphs, which are texts describing processes. It contains 488 paragraphs and 3,300 sentences describing 183 processes. Each paragraph is richly annotated with the existence (created or destroyed) and locations (whether motion has occurred, of what entity, and to what destination) of all the main entities at every time step (sentence) throughout the procedure (∽81,000 annotations). It has an 80/10/10 data split, and it is ensured that the test paragraphs are unseen in train and dev.

We evaluated Lexis on the ProPara dataset in three experimental settings. In the first setting, Lexis utilized only the SemParse-instantiated VerbNet semantic representations and achieved an F1 score of 33%. In the second setting, Lexis was augmented with the PropBank parse and achieved an F1 score of 38%. An error analysis suggested that in many cases Lexis had correctly identified a changed state but that the ProPara data had not annotated it as such, possibly resulting in misleading F1 scores. For this reason, Kazeminejad et al., 2021 also introduced a third “relaxed” setting, in which the false positives were not counted if and only if they were judged by human annotators to be reasonable predictions. To accomplish that, a human judgment task was set up and the judges were presented with a sentence and the entities in that sentence for which Lexis had predicted a created, destroyed, or moved state change, along with the locus of state change. The results were compared against the ground truth of the ProPara test data. If a prediction was incorrectly counted as a false positive, i.e., if the human judges counted the Lexis prediction as correct but it was not labeled in ProPara, the data point was ignored in the evaluation in the relaxed setting. This increased the F1 score to 55% – an increase of 17 percentage points.

An error analysis of the results indicated that world knowledge and common sense reasoning were the main sources of error, where Lexis failed to predict entity state changes. An example is in the sentence “The water over the years carves through the rock,” for which ProPara human annotators have indicated that the entity “*space”* has been CREATED. This is extra-linguistic information that is derived through world knowledge only. Lexis, and any system that relies on linguistic cues only, is not expected to be able to make this type of analysis. It is important to recognize the border between linguistic and extra-linguistic semantic information, and how well VerbNet semantic representations enable us to achieve an in-depth linguistic semantic analysis.

## 5. Discussion

Despite impressive advances in NLU using deep learning techniques, human-like semantic abilities in AI remain out of reach. The brittleness of deep learning systems is revealed in their inability to generalize to new domains and their reliance on massive amounts of data—much more than human beings need—to become fluent in a language. The idea of directly incorporating linguistic knowledge into these systems is being explored in several ways. Our effort to contribute to this goal has been to supply a large repository of semantic representations linked to the syntactic structures and classes of verbs in VerbNet. Although VerbNet has been successfully used in NLP in many ways, its original semantic representations had rarely been incorporated into NLP systems (Zaenen et al., 2008; Narayan-Chen et al., 2017). We have described here our extensive revisions of those representations using the Dynamic Event Model of the Generative Lexicon, which we believe has made them more expressive and potentially more useful for natural language understanding.

As in any area where theory meets practice, we were forced to stretch our initial formulations to accommodate many variations we had not first anticipated. VerbNet is a large, domain-independent resource of English verbs. Although its coverage of English vocabulary is not complete, it does include over 6,600 verb senses. We were not allowed to cherry-pick examples for our semantic patterns; they had to apply to every verb and every syntactic variation in all VerbNet classes.

We strove to be as explicit in the semantic designations as possible while still ensuring that any entailments asserted by the representations applied to all verbs in a class. Occasionally this meant omitting nuances from the representation that would have reflected the meaning of most verbs in a class. It also meant that classes with a clear semantic characteristic, such as the type of emotion of the Experiencer in the admire-31.2 class, could only generically refer to this characteristic, leaving unexpressed the specific value of that characteristic for each verb.

Recently, Kazeminejad et al. (2022) has added verb-specific features to many of the VerbNet classes, offering an opportunity to capture this information in the semantic representations. These features, which attach specific values to verbs in a class, essentially subdivide the classes into more specific, semantically coherent subclasses. For example, verbs in the admire-31.2 class, which range from *loathe* and *dread* to *adore* and *exalt*, have been assigned a +negative_feeling or +positive_feeling attribute, as applicable.

We have begun experimenting with incorporating the features into our semantic representations to further increase their expressive power. The admire-31.2 class, for example, includes the predicate **has_emotional_state**(E, Experiencer, V_Emotion), in which the feature value for a specific verb can replace V_Emotion when instantiating the representation from actual text. Likewise, in the calibratable_cos-45.6.1 class, the predicate **change_value** includes the argument V_Direction, whose value can be found in context from a particular verb's verb-specific feature: either increase (e.g., *rise*), decrease (e.g., *fall*) or fluctuate (e.g., *vary*).

(29)    GL-Verbnet representation
              *The price of oil rose by 500% from $5 to $25*.
              **has_attribute**(E, *oil*_Patient, *price*_Attribute)
              **has_val**(*e*_1_, *oil*_Patient, *$5*_Initial_State)
              **change_value**(*e*_2_, increase_V_Direction, *500%*_Extent,
              *price*_Attribute, *oil*_Patient)
              **has_val**(*e*_3_, *oil*_Patient, *$25*_Result)

We are exploring how to add slots for other new features in a class's representations. Some already have roles or constants that could accommodate feature values, such as the admire class did with its Emotion constant. We are also working in the opposite direction, using our representations as inspiration for additional features for some classes. The compel-59.1 class, for example, now has a manner predicate, with a V_Manner role that could be replaced with a verb-specific value. The verbs of the class split primarily between verbs with a compel connotation of compelling (e.g., *oblige, impel*) and verbs with connotation of persuasion (e.g., *sway, convince*) These verbs could be assigned a +compel or +persuade value, respectively.

The true test of this resource will be in its usefulness to the field. We are encouraged by the efficacy of the semantic representations in tracking entity changes in state and location. We would like to see if the use of specific predicates or the whole representations can be integrated with deep-learning techniques to improve tasks that require rich semantic interpretations.

## Data Availability Statement

The datasets presented in this study can be found in online repositories. The names of the repository/repositories and accession number(s) can be found at: VerbNet GitHub https://github.com/cu-clear/verbnet.

## Author Contributions

SB led the effort. SB and JB did the work and wrote the majority of the paper. GK did the evaluation and wrote that section. JP, AZ, and MP provided input and feedback over several years of conference calls.

## Funding

We gratefully acknowledge the support of DTRAl-16-1-0002/Project # 1553695, eTASC-Empirical Evidence for a Theoretical Approach to Semantic Components; DARPA 15-18-CwC-FP-032 Communicating with Computers, C3 Cognitively Coherent Human-Computer Communication (sub from UIUC) and Elementary Composable Ideas (ECI) Repository (sub from SIFT); DARPA KAIROS Program No. FA8750-19-2-1004; and DARPA FA8750-18-2-0016-AIDA—RAMFIS: Representations of vectors and Abstract Meanings for Information Synthesis. Any opinions, findings, and conclusions or recommendations expressed in this material are those of the authors and do not necessarily reflect the views of DTRA or the U.S. government.

## Conflict of Interest

The authors declare that the research was conducted in the absence of any commercial or financial relationships that could be construed as a potential conflict of interest.

## Publisher's Note

All claims expressed in this article are solely those of the authors and do not necessarily represent those of their affiliated organizations, or those of the publisher, the editors and the reviewers. Any product that may be evaluated in this article, or claim that may be made by its manufacturer, is not guaranteed or endorsed by the publisher.

## Footnotes

^1^In VerbNet semantic representations-both old and new-any thematic role that appears in other representations of the class but that is not instantiated in the current syntactic frame is prefixed with a question mark (see ?Initial_location in 1).

^2^All the examples in this section are from the ProPara dataset (Dalvi et al., 2018).
