# Supplementary material for: Semantic Representations for NLP Using VerbNet and the Generative Lexicon
Source: Front Artif Intell. 2022 Apr 14;5:821697. doi: 10.3389/frai.2022.821697 (PMC9048683; doi:10.3389/frai.2022.821697)
Supplement: Supplementary file 1 [file Table_1.DOCX]

| **Appendix A: Predicate List** | | | | | | |
| --- | --- | --- | --- | --- | --- | --- |
| **predicate name** | **definition** | **slot1** | **slot2** | **slot3** | **slot4** | **slot5** |
| **abide_by** | An agent holds an ideology or belief. | time stamp: E, e | follower: Agent | ideology: Theme |  |  |
| **about** | A communication or social interaction event has a Theme as a topic or concern. | subevent:  e | subject matter: Theme |  |  |  |
| **act** | A participant carries out the action or serves the function described in the last argument slot. | time stamp: e | actor: Agent Recipient Patient | act: Eventuality Result V_Attribute Attribute |  |  |
| **adjusted** | A Patient is in a state of being adjusted to some Goal. Subtype of has_state. | time stamp: e | adjustor:  Patient | adjusted to: Goal |  |  |
| **admit** | An Agent allows an entity Theme to enter a Location. | time stamp: e | admitter: Agent | admittee: Theme | admitted to: Location |  |
| **alive** | An animate Patient is alive. Subtype of has_state. | time stamp: e (stative) | liver: Patient |  |  |  |
| **allow** | A Causer allows a Beneficiary to engage in an activity (Eventuality). | time stamp: e | allower: Causer Agent | allowed: Eventuality | entity given permission: Beneficiary |  |
| **appear** | A Theme appears or materializes. | time stamp: e | appearer: Theme |  |  |  |
| **apply_heat** | Using an Instrument, heat is applied to a Patient. | time stamp:  ë | heating implement: Instrument | heated entity: Patient |  |  |
| **apply_material** | An Agent applies a material to a Patient. | time stamp:  ë | surface: Patient | material: V_Material |  |  |
| **approve** | An animate agent or organization approves a Theme. | time stamp: e | approver: Agent | approvee: Theme |  |  |
| **assess** | An animate Agent or organization assesses a Theme to determine its Value. | time stamp: e | assessor: Agent | assessed: Theme Theme_I/J Co-Theme | for what: Attribute | value: Value (PS) |
| **attached** | Two (or more) Patients are physically attached to one another. | time stamp: e (stative) | attachee1: Patient Patient_I | attachee2: Co-Patient Patient_J |  |  |
| **attempt** | An animate Agent or organization engages in an Eventuality aiming for a certain result state, with or without success. | time stamp: ë | tryer: Agent | tried: Eventuality |  |  |
| **authority_**  **relationship** | Two participants have a relationship (in the situation or in general) in which one has more control, either over the other or over some element of the situation. | time stamp: e, E, ë | authority figure: Agent Beneficiary Pivot Recipient | subordinate: Co-Agent Agent Theme |  |  |
| **avoid** | An animate Agent avoids a Theme or Location | time stamp: e | avoider: Agent | avoidee: Theme Source Location |  |  |
| **be** | An entity exists. | time stamp: e, E | entity that exists: Patient Product Result Precondition |  |  |  |
| **become** | One entity transforms into another entity | time stamp: e | start entity: Patient | end entity: Product |  |  |
| **believe** | An animate Agent or organization believes a Theme to be in some event or state. | time stamp: E | believer: Agent | entity subject to belief: Theme | belief held regarding the Theme: Eventuality |  |
| **benefit** | A Beneficiary benefits from some event. | time stamp: e | beneficiary: Beneficary |  |  |  |
| **body_motion** | An Agent performs an action or motion with a part of his/her body. | time stamp: ë | mover: Agent |  |  |  |
| **body_process** | An animate Agent undergoes a bodily process. | time stamp: ë | entity with a body: Agent |  |  |  |
| **body_reflex** | An animate Experiencer undergoes a bodily reflex, possibly in response to a Stimulus. | time stamp: ë, e | entity with a body: Experiencer |  |  |  |
| **body_sensation** | An animate Experiencer feels a sensation in their body | time stamp: e | entity with a body: Experiencer |  |  |  |
| **calculate** | An animate Agent or organization performs a mathematical operation on two operands (a plural Theme, or a Theme and Co-Theme). | time stamp: e | calculator: Agent | first operand: Theme | second operand: Co-Theme |  |
| **cause** | One event causes another event to occur. | causing event: ë, e | caused event: ë, e |  |  |  |
| **change_value** | A Patient moves along a scale in some Direction to attain a new value. | time stamp: e | entity with value: Patient | value: Initial_State Result |  |  |
| **characterize** | An animate Agent or organization has an opinion about a Theme as having an Attribute, and possibly expresses that opinion. | time stamp: e, E | characterizer: Agent | characterized: Theme | characteristic: Attribute |  |
| **charge** | A human or organization Agent apprehends or puts a human Patient through the legal system for some crime/Theme. | time stamp: e | authority: Agent | criminal: Patient | crime: Theme |  |
| **co-temporal** | Two subevents occur fully at the same time. | first event: ë, e | second event: ë, e |  |  |  |
| **completed** | A natural terminus is reached. | time stamp: e | thing completed: Eventuality |  |  |  |
| **conclude** | An animate Agent or organization reaches a conclusion. | time stamp: e | concluder: Agent | conclusion: Theme |  |  |
| **confined** | A Theme is confined. | time stamp: e (stative) | entity confined: Theme |  |  |  |
| **conflict** | An Agent and Co-Agent (animate or organization) engage in conflict. A subtype of social_interaction. | time stamp: e (stative) | first entity in conflict: Agent Agent_i | second entity in conflict: Patient Co-Agent Agent_j |  |  |
| **contact** | Two participants come into physical contact. | time stamp: e, E, ë | first entity in contact: Agent Theme  Theme_i Patient Patient_i  Instrument | second entity in contact: Theme Co-Theme Theme_j Patient Patient_j Instrument Location Experiencer |  |  |
| **contain** | A Pivot contains a Theme | time stamp: E | container: Pivot | contained: Theme |  |  |
| **control** | An Agent has control over a Theme | time stamp: e | controller: Agent | controllee: Theme |  |  |
| **cooked** | A Patient has been cooked into the final state described by the verb | time stamp: e (stative) | cooked entity: Patient | final state: V_Final_State |  |  |
| **cooperate** | An animate Agent or organization cooperates with another animate or organizational Agent/Co-Agent on some Theme. | time stamp: ë | first cooperator: Agent Agent_i | second cooperator: Co-Agent Agent_j | subject matter: Theme |  |
| **correlated** | A Theme and Co-Theme correlate or alternate with each other. | time stamp: E, e (stative) | first entity: Theme | second entity: Co-Theme |  |  |
| **cost** | The cost of an event or or item is equal to a monetary Asset. | time stamp: E | valuable entity: Product Theme | price: Asset |  |  |
| **covered** | Patient is in a state of being covered by a Material | Time stamp: e (stative) | covered entity: Patient | cover, substance: V_Material |  |  |
| **create_image** | An Agent creates an image of some Theme. | Time stamp: e | creator: Agent | creation: Theme |  |  |
| **declare** | An animate Agent or organization makes a judgment and declaration about a Theme as having an Attribute. There isn't necessarily a transfer of information to an audience (during the topic time of the situation) associated with the declaration | Time stamp: e | declarer: Agent | entity judged: Theme Patient | what is declared about them: Attribute Result |  |
| **dedicate** | An animate or organizational Agent dedicates a Theme, often reciprocal, to a Goal. | Time stamp: e | dedicator: Agent | dedicated what: Theme | to what: Goal |  |
| **degraded** | A Patient or Co-Patient's wholeness is broken down. | Time stamp: e (stative) | physical entity: Patient |  |  |  |
| **depend** | An Agent depends on a Theme. | Time stamp: E | dependant: Agent | depended on: Theme |  |  |
| **desire** | An entity desires or needs a Theme or Stimulus. | Time stamp: e (stative), E | wanter: Patient Pivot | wanted: Theme |  |  |
| **destroyed** | An Agent destroys a concrete Patient. | Time stamp: e (stative) | entity destroyed: Patient |  |  |  |
| **develop** | A Patient develops into a more mature form. | time stamp: ë | developing entity: Patient Product |  |  |  |
| **differ** | A Theme and a Co-Theme are different. | Time stamp: E | first entity: Theme Theme_i | second entity: Co-Theme Theme_j | domain of difference: Attribute (pred  Specific) |  |
| **disappear** | A Theme disappears--changes from being at a location to not being at that location or from visible to not visible. | time stamp: e | disappearing entity: Theme |  |  |  |
| **discomfort** | A body part Patient experiences discomfort. | time stamp: ë | experiencer: Patient Experiencer |  |  |  |
| **discourage** | A Causer discourages a Beneficiary from engaging in an Eventuality. | time stamp: e | discourager: Causer | discouraged action: Eventuality | dis-couraged actor: Beneficiary |  |
| **do** | An agent does something unspecified. This action often causes something else later in the representation. | time stamp: e | doer: Agent |  |  |  |
| **duration** | The run time of the representation lasts for a certain Duration. | subevent: e | duration: Duration |  |  |  |
| **earn** | A Theme earns an Asset. | time stamp: e | earner: Theme | earned: Asset |  |  |
| **elliptical_**  **motion** | An Agent or a Theme is in motion in such a way that an elliptical trajectory is created around an Axis. The Axis is not part of or topologically internal to the entity in motion. | time stamp: ë | entity in motion: Theme | axis: Axis |  |  |
| **emit** | A participant produces and sends out an emission that may serve as sensory input. | time stamp: ë, e | emitter: Agent Theme | emitted: V_Sound Theme V_Odor |  |  |
| **encourage** | An Agent encourages (but does not force) ... | time stamp: e | encourager: Agent | encouraged activity: Theme |  |  |
| **endangered** | A participant is in danger. | time stamp: e (stative) | entity in danger: Beneficiary Pivot | dangerous thing: Theme |  |  |
| **endure** | A Theme undergoes or experiences some Circumstance. | time stamp: e | undergoer: Theme | circumstance: Circumstance |  |  |
| **engage_in** | A participant engages in an activity. | time stamp: ë, e | actor: Agent Affector | activity: Eventuality Subeventuality Theme V_Theme V_Eventuality |  |  |
| **ensure** | A Theme is ensured. | time stamp:  E | ensurer: Precondition | ensured: Theme |  |  |
| **equals** | Two roles in the representation are filled by the same participant. | first argument: Agent Recipient Topic Agent Destina-tion V_Form Recipient Initial_  Location Topic Theme | second argument: Patient Stimulus Eventuality Source Axis / Trjaectory Trajectory Stimulus Destination Eventuality Trajectory |  |  |  |
| **exceed** | A Pivot exceeds a Theme in terms of some Attribute | time stamp: e | entity with more: Pivot | entity with less: Theme | domain: Attribute |  |
| **exert_force** | An Affector exerts force on a Theme | time stamp: e | actor: Agent | undergoer: Theme Instrument |  |  |
| **fictive_motion** | A Theme has a physical form that mimics a motion Trajectory. | time stamp: E | thing with a form: Theme | motion-path-like form: Trajectory |  |  |
| **financial_**  **interest_in** | A Goal has a property known as financial_interest, which is a transferable possession. | the financial interest itself: Theme | financial interest in what entity/ organization: Goal |  |  |  |
| **financial_**  **interaction** | Two entities (Agent and Recipient) engage in an underspecified financial interaction regarding a sum (Asset). | time stamp:  e | first entity in relationship: Agent | second entity: Recipient | money: Asset |  |
| **find** | An Agent finds a sought-for entity. | time stamp:  e | finder: Agent | found: Theme |  |  |
| **finish** | A subevent finishes a larger event, meaning it is the last portion of the larger event, and brings the larger event to an end. | subevent: e | event: ë |  |  |  |
| **free** | A Source no longer contains or experiences a Theme. | time stamp: e (stative) | potential confiner: Source | entity with liberty: Theme |  |  |
| **full_of** | A Location has a capacity which is met by its containment of a Theme. | subevent: E | container with capacity: Location | contents: Theme |  |  |
| **function** | A Theme fulfills or performs some function. | time stamp: e | entity with a function: Theme Patient |  |  |  |
| **give_birth** | An Agent gives birth to a Patient. | time stamp: e | mother: Agent | baby: V_Patient Patient |  |  |
| **handle** | An Agent grapples with a potentially challenging situation (Theme). | time stamp:  ë, e | handler: Agent | handled: Theme |  |  |
| **harm** | An Agent engages in a process or accomplishment that affects a Maleficiary for the worse. | time stamp: ë, e | harmer: Agent | harmed: Maleficiary |  |  |
| **harmed** | A Patient undergoes a change of state and ends up harmed in some way (may or may not be physical). | time stamp: e (stative) | harmed: Patient |  |  |  |
| **harmonize** | A Theme and Co-Theme are paired well. | time stamp:  E | first entity: Theme | second entity: Co-Theme |  |  |
| **has_attribute** | A participant has an Attribute | time stamp: E, e (stative) | entity with attribute: Theme Patient Stimulus Result Recipient | attribute: Attribute Importance (constant) |  |  |
| **has_boundary** | a Theme has a boundary at a Location | time stamp: E | entity with boundary: Theme | the boundary: Location |  |  |
| **has_capacity** | A Location has a capacity/can contain a certain amount equal to some Value. | time stamp: E | entity with a capacity: Location | capacity: Value |  |  |
| **has_**  **configuration** | A Theme is in a spatial Configuration (may refer to physical_form, position, and/or orientation). | time stamp: e (stative) | entity in a configuration: Theme | the configuration: V_  Configuration |  |  |
| **has_**  **designation** | A Theme or Patient has a name, attribute or official designation | time stamp: e (stative) | designated entity: Theme | designation: Result V_Result |  |  |
| **has_emotional_**  **state** | A participant experiences an emotional state. | time stamp: E, e (stative)) | emotional entity: Patient Experiencer | emotion: V_Final_State V_Emotion  Constants: - negative_  emotion - deceived |  |  |
| **has_information** | An entity possesses information. | time stamp: e (stative), E | info-haver: Agent Agent_I Agent_J Co-Agent Recipient Source | info: Topic Topic_I Topic_J Theme Source |  |  |
| **has_location** | A participant is in a Location. | time stamp: E e | located entity: Agent Co-Agent Theme Co-Theme Patient V_Odor | the location: Location Source Goal |  |  |
| **has_material_**  **integrity_state** | A physical entity has material integrity in some state. | time stamp: e (stative) | material entity: Patient Patient_I Patient_J Co-Patient | integrity state: V_State |  |  |
| **has_**  **organization_**  **role** | An entity works for an organization in a particular role | time stamp: e (stative) | worker: Agent Theme Pivot | role: Attribute | organization: Beneficiary Source Goal |  |
| **has_orientation** | An entity is oriented in relation to a space or framework. | time stamp: e (stative) | spatial entity: Theme | orientation: V_Orientation |  |  |
| **has_physical_**  **form** | As the Result of an event, an Undergoer has some new physical form. | time-stamp: e (stative) E | entity with a form: Patient Theme | the form: V_Final_State V_Form (predspecific) |  |  |
| **has_position** | An animate entity is in a posture. | time stamp: e (stative) | posed entity: Agent Patient Experiencer | posture: V_Position Position (predspecific) |  |  |
| **has_possession** | A Participant has possession of or control over a Theme or Asset | time stamp: E, e (stative) | possessor: Agent  Agent_I  Agent_J  Co-Agent  Goal  Pivot  Recipient  Source  Theme | possession: Asset  Co-Theme  Source  Theme  Theme_I  Theme_J  Theme"  V_Theme |  |  |
| **has_role** | A participant serves the role named by the Attribute | time stamp: E | actor: Agent Recipient | role: V_Attribute Attribute |  |  |
| **has_sentiment** | A participant has a sentiment | time stamp: e (stative) | feeler: Agent | sentiment: V_Attribute |  |  |
| **has_set_**  **member** | a set (Goal or Pivot) has a member Theme. | time stamp: E e (stative) | set: Pivot Goal | member: Theme |  |  |
| **has_spatial_**  **relationship** | A Theme and Co-Theme are in a spatial relationship with one another as specified by the verb. | time stamp: E | first entity: Theme | second entity: Co-Theme | spatial relation: V_Spatial_Relation |  |
| **has_state** | A Patient or Material is in some State | time stamp: e (stative) | entity in state: Experiencer Patient Source Material Theme Product InitialLocation | the state: Result V_Final_State Initial_State Stimulated (constant) |  |  |
| **has_temporal_**  **location** | An event is anchored to a time. | time  stamp: e (stative) | event: Eventuality | time: Destination_  Time (pred-specific) |  |  |
| **has_value** | A Patient has a value. | time stamp: e (stative) | entity with value: Patient | value: Initial_State Result |  |  |
| **help** | An Agent helps a Beneficiary with some endeavor Theme. | time stamp: e (stative) | helper: Agent | helpee: Beneficiary | helped with: Theme |  |
| **in_reaction_to** | The subevent in question occurs in reaction to a Stimulus | subevent induced: E e ë (inherited) | Stimulus |  |  |  |
| **indicate** | One proposition points to another, perhaps as a prediction, an explanation, an implication, or support. | time stamp: E | indicator: Pivot | indicated: Topic |  |  |
| **injury** | A Patient undergoes an injury event of the type indicated by the verb. | time stamp: e | injured entity: Patient | injury type: V_Injury |  |  |
| **intend** | An Agent intends something. | time stamp: E | intender: Agent | intention: Topic |  |  |
| **intrinsic_motion** | an entity is moving; this kind of motion does not entail change of location, but rather describes movement of parts | time stamp: ë | entity in motion: Theme | trajectory: Trajectory (PredSpecific) |  |  |
| **involuntary** | A participant has an involuntary bodily reaction. | time stamp: ë | entity acting involuntarily: Experiencer |  |  |  |
| **involved** | A Theme is involved in some Goal situation | time stamp: e (stative) | entity involved: Theme | involved in what: Goal |  |  |
| **irrealis** | a subevent is expected, but not confirmed | irrealis event: e ë E |  |  |  |  |
| **judge** | An Agent completes the act of making a judgment (Attribute) about a Theme. | time stamp: e | judge: Agent | entity judged: Theme | judgement: Attribute |  |
| **limit** | A Causer limits a Patient to some Goal. | time stamp: e | causer of limit: Causer | entity subject to limit: Patient | the limit: Goal |  |
| **made_of** | A Product consists of a Material. | time stamp: e (stative) | thing made of something: Product | the material: Material |  |  |
| **manner** | A participant is involved in a situation according to a certain Manner. | time stamp: ë e E | **Constant:** aggressive deceptive directedmotion enticing forceful hostile illegal movement playful quality stimulating unserious  **VerbSpecific:** V_Manner  **Primary Role:** Manner | **entity having a manner:**  Agent Co-Agent Theme Co-Theme Instrument Causer |  |  |
| **meets** | Event 0 and Event 1 intersect (temporally). | first event: e | second event: e |  |  |  |
| **mingled** | multiple participants have their constituent elements mixed together | time stamp: e (stative) | first entity: Patient Patient_i | second entity: Co-Patient Patient_j |  |  |
| **motion** | An entity is in motion. | time stamp: ë | entity in motion: Theme | trajectory: Trajectory |  |  |
| **necessitate** | A Theme necessitates that some Precondition be met | time stamp: E | needer: Theme | needed: Precondition |  |  |
| **occur** | An Eventuality occurs. | time stamp: ë e | thing occurring: Eventuality |  |  |  |
| **operate_vehicle** | A participant operates a vehicle. | time stamp: ë | driver: Theme | vehicle: V_Vehicle |  |  |
| **opposition** | Two roles are in opposition to each other. | first entity: Initial_State | second entity: Result |  |  |  |
| **overlaps** | two subevents overlap temporally | subevent 1: ë e | subevent 2:  ë  e |  |  |  |
| **pace** | An event occurs at a particular Pace | event with a pace: ë  e | the pace: **Constant:** Slow Fast |  |  |  |
| **part_of** | One entity is a part of another entity, physically or abstractly. | part entity: Theme Patient V_Theme Location | whole entity: Agent Source Experiencer Destination Patient |  |  |  |
| **penetrating** | An Instrument penetrates into and possibly passes completely through a Patient. | time-stamp: e (stative) | penetrator: Instrument | penetrated: Patient |  |  |
| **perceive** | A Participant preceives a Stimulus or Theme. | time-stamp: e (full range) E | perceiver: Experiencer | perceived: Stimulus Topic |  |  |
| **perform** | An Agent gives a performance of some Theme. | time stamp: ë | performer: Agent | performed: Theme |  |  |
| **procreate** | A (frequently plural) Agent procreates. | time stamp: e | entity procreating: Agent |  |  |  |
| **relate** | A Theme relates/pertains to a Co-Theme. | time stamp: E | first entity involved in the relation: Theme | second entity involved in the relation: Co-Theme |  |  |
| **repeated_**  **sequence** | A sequence of subevents repeats. | first subevent e | second subevent: e | third subevent: e | fourth subevent: e | etc. |
| **require** | A Theme requires that a Precondition be met by some Source. | time stamp: E | needer: Theme | needed: Precondition | needed from: Source |  |
| **reside** | A Theme is in residence in a Location. | time stamp:  ë | entity residing: Theme | residence: Location |  |  |
| **rotational_**  **motion** | A Theme undergoes a change in orientation. | time stamp:  ë | entity in motion: Theme | trajectory: Trajectory | amount rotated: Extent |  |
| **satisfy** | A Pivot satisfies a requirement (Theme). | time stamp: E, e | satisfier: Pivot | requirement: Theme |  |  |
| **search** | An Agent searches a Place for a Theme. | time stamp: e | searcher: Agent | location searched: Location Source | thing sought: V_Theme Theme |  |
| **seem** | A Theme seems to be some Attribute. | time stamp: E | seemer: Theme | attribute: Attribute |  |  |
| **signify** | A Theme represents a Co-Theme in some Context. | time stamp: E | sign: Pivot | signified: Co-Theme | in what context: Attribute |  |
| **sleep** | An Agent sleeps. | time stamp:  ë | sleeper: Agent |  |  |  |
| **social_**  **interaction** | An Agent and a Co-Agent interact socially. | time stamp:  ë e | first interactor: Agent Agent_I | second interactor: Co-Agent Agent_J |  |  |
| **spend** | An Agent spends an Asset on a Goal. | time stamp: e | spender: Agent | money: Asset | purchase: Goal |  |
| **spend_time** | An Agent engages in an Eventuality for a fixed Duration. | time stamp: e | Entity spending time: Agent | on what activity: Eventuality |  |  |
| **start** | one subevent is the beginning portion of another subevent. | first subevent: e | second subevent: ë |  |  |  |
| **subjugated** | A Patient is subjugated or repressed. | time stamp: e (stative) | subjugated entity: Patient |  |  |  |
| **succeed** | An Agent succeeds or fails at some activity or Theme. | time stamp: e | successful (or not) entity: Agent | endeavor: Eventuality |  |  |
| **suffocated** | An animate Theme is suffocated. | time stamp: e (stative) | entity suffocated: Patient | final state: V_Final_State |  |  |
| **support** | One participant is either physical or informational support for another participant | time stamp: E | supporting entity: Source Pivot | supported: Theme |  |  |
| **suspect** | An Agent believes and possibly acts on the idea that a Theme is guilty of an Attribute. | time stamp: e | suspector: Agent | entity suspected of something: Theme | the something they are suspected of: Attribute |  |
| **take_care_of** | An Agent takes care of a Patient, Theme, or Product. | time stamp:  e ë | care-giver: Agent | cared for: Patient Theme Product |  |  |
| **temporal_**  **motion** | An event that was scheduled for one point in time is moved to another point in time. | time stamp:  ë | eventuality scheduled: Eventuality | moved in which temporal direction: Trajectory (predSpecific) |  |  |
| **think** | An animate or ogranization Agent considers a Theme | time stamp: E | considerer: Agent | considered: Theme Topic |  |  |
| **together** | Two Undergoers are physically together. | time stamp: e (stative) E ë | first entity included: Patient Patien_I Theme Theme_I | second entity included: Co-Patient Patient_J Co-Theme Theme_J |  |  |
| **transfer** | Possession or control over a Theme is transfered from one participant to another. | time stamp: e | giver: Agent Co-Agent Recipient Goal Source | item transfered: Theme V_Theme Co-Theme Asset | recipient: Location Source Recipient Agent Co-Agent Goal |  |
| **transfer_info** | One participant transfers some information/Topic to (or from) another participant. | time stamp: e | giver/  communicator: Agent Recipient Co-Agent Source | information: Theme Topic | recipient/  hearer: Agent Recipient Co-Agent Source |  |
| **understand** | An Experiencer has information (Stimulus) and has integrated it into understanding. | time stamp:  E | understander: Experiencer | understood: Stimulus |  |  |
| **use** | An Agent uses a Theme for the purpose of accomplishing some Eventuality. | time stamp: e | user: Agent | used: Theme | purpose: Eventuality |  |
| **utilize** | An Agent utilizes something. | time stamp: e | user: Agent | used: Source Theme Instrument |  |  |
| **visible** | A Participant is visible. | time-stamp: e (stative) | visible entity: Patient |  |  |  |
| **voided** | A situation or entity becomes voided in a specific manner inidicated by the verb. | time-stamp: e (stative) | entity voided: Patient | particular state of void: V_Final_State |  |  |
| **wear** | An Agent wears an item of clothing/Theme. | time stamp:  ë e | wearer: Agent Pivot | worn: Theme |  |  |
| **weather** | A weather event occurs, possibly precipitating some entity/Theme. | time stamp:  ë | Weather type: V_Weather | cognate object: Theme |  |  |
| **work** | An agent works on a job. | time stamp: e,  ë | worker: Agent | job: Theme |  |  |
| **yield** | An Agent yields to a Co-Agent or Theme. | time stamp: e | yielder: Agent | yielded to: Pivot | on what issue: Theme |  |
