# Supplementary material for: Semantic Representations for NLP Using VerbNet and the Generative Lexicon
Source: Front Artif Intell. 2022 Apr 14;5:821697. doi: 10.3389/frai.2022.821697 (PMC9048683; doi:10.3389/frai.2022.821697)
Supplement: Supplementary file 2 [file Table_2.DOCX]

**Appendix B: Predicates Organized by Aspect**

| **Situation predicates** | | | |
| --- | --- | --- | --- |
|  | **State**- holds as true at every point during the phase named | | |
|  | 67 | Be() |  |
|  |  | Has_possession() |  |
|  |  | Conflict() |  |
|  |  | Harmed() |  |
|  |  | Understand() |  |
|  |  | Together() |  |
|  |  | Abide_by() |  |
|  |  | Adjusted() |  |
|  |  | Contain() |  |
|  |  | Cooked() |  |
|  |  | Correlated() |  |
|  |  | Cost() |  |
|  |  | Covered() |  |
|  |  | Degradation_material_integrity() |  |
|  |  | Depend() |  |
|  |  | Desire() |  |
|  |  | Destroyed() |  |
|  |  | Differ() |  |
|  |  | Endangered() |  |
|  |  | Ensure() |  |
|  |  | Fictive_motion() |  |
|  |  | Free() |  |
|  |  | Full_of() |  |
|  |  | Has_attribute() |  |
|  |  | Has_configuration() |  |
|  |  | Has_designation() |  |
|  |  | Has_emotional_state() |  |
|  |  | Has_information() |  |
|  |  | Has_location() |  |
|  |  | Has_material_integrity_state() |  |
|  |  | Has_position() |  |
|  |  | Has_role() |  |
|  |  | Has_sentiment() |  |
|  |  | Has_set_member() |  |
|  |  | Has_state() |  |
|  |  | Has_temporal_location() |  |
|  |  | Has_value() |  |
|  |  | Help() |  |
|  |  | Indicate() |  |
|  |  | Intend() |  |
|  |  | Made_of() |  |
|  |  | Penetrating() |  |
|  |  | Seem() |  |
|  |  | Signify() |  |
|  |  | Visible() |  |
|  |  | Voided() |  |
|  |  | Alive() |  |
|  |  | Attached() |  |
|  |  | Believe() |  |
|  |  | Has_capacity() |  |
|  |  | Confined() |  |
|  |  | Harmonize() |  |
|  |  | Has_boundary() |  |
|  |  | Has_orientation() |  |
|  |  | Has_spatial_relationship() |  |
|  |  | Involved() |  |
|  |  | Mingled() |  |
|  |  | Necessitate() |  |
|  |  | Has_physical_form() |  |
|  |  | Relate() |  |
|  |  | Require() |  |
|  |  | Subjugated() |  |
|  |  | Suffocated() |  |
|  |  | Support() |  |
|  |  | Has_organization_role() |  |
|  |  | Authority_relationship() |  |
|  |  | Contact() |  |
|  |  | Be() |  |
|  |  |  |  |
|  | **Perfective** (event comes to an end, either an accomplishment or achievement) | | |
|  | 14 | Attempt() |  |
|  |  | Earn() |  |
|  |  | Injury() |  |
|  |  | Financial_interaction() |  |
|  |  | Give_birth() |  |
|  |  | Transfer() |  |
|  |  | Transfer_info() |  |
|  |  | Yield() |  |
|  |  | Become() |  |
|  |  | Calculate() |  |
|  |  | Change_value() |  |
|  |  | Do() |  |
|  |  | Procreate() |  |
|  |  | spend() |  |
|  |  |  |  |
|  |  | **Accomplishment** (includes a process, a culmination and result state) | |
|  |  | 7 | Charge() |
|  |  |  | Conclude() |
|  |  |  | Create_image() |
|  |  |  | Declare() |
|  |  |  | Endure() |
|  |  |  | Judge() |
|  |  |  | Spend_time() |
|  |  |  |  |
|  |  | **Achievement** (is the transition between a start state and a result state) | |
|  |  | 4 | Disappear() |
|  |  |  | Find() |
|  |  |  | Succeed() |
|  |  |  | Appear() |
|  |  |  |  |
|  | **Process** (durative transition, does not come to an end within topic time of rep) | | |
|  | 20 | Develop() |  |
|  |  | Discomfort() |  |
|  |  | Elliptical_motion() |  |
|  |  | Intrinsic_motion() |  |
|  |  | take_care_of() |  |
|  |  | Temporal_motion() |  |
|  |  | Rotational_motion() |  |
|  |  | Apply_heat() |  |
|  |  | Apply_material() |  |
|  |  | Body_motion() |  |
|  |  | Body_process() |  |
|  |  | Body_reflex() |  |
|  |  | Body_sensation() |  |
|  |  | Cooperate() |  |
|  |  | Motion() |  |
|  |  | Perform() |  |
|  |  | Reside() |  |
|  |  | Sleep() |  |
|  |  | Operate_vehicle() |  |
|  |  | Work() |  |
|  |  | Function() |  |
|  | **Process/Perfective** (some verbs make these predicates processes only, some make them culminate) | | |
|  | 10 | Emit() |  |
|  |  | Engage_in() |  |
|  |  | Search() |  |
|  |  | Social_interaction() |  |
|  |  | Assess() |  |
|  |  | Dedicate() |  |
|  |  | Occur() |  |
|  |  | Use() |  |
|  |  | Exert_force() |  |
|  |  | Act() |  |
|  |  |  |  |
|  | **Process/Accomplishment** (some verbs make these predicates processes only, some make them culminate with result state) | | |
|  | 2 | Handle() |  |
|  |  | Harm() |  |
|  |  |  |  |
|  | **Process/State** (verbs may be construed as either states or processes, or some verbs are one and some are the other) | | |
|  | 5 | Wear() |  |
|  |  | Perceive() |  |
|  |  | Think() |  |
|  |  |  |  |
|  | **Process/State/Perfective** (verbs may be used as any of the 3) | | |
|  | 6 | Suspect() |  |
|  |  | Encourage() |  |
|  |  | Approve() |  |
|  |  | Avoid() |  |
|  |  | Control() |  |
|  |  | Discourage() |  |
|  |  |  |  |
|  | **State/Perfective** | | |
|  | 5 | Characterize() |  |
|  |  | Satisfy() |  |
|  |  | Exceed() |  |
|  |  | Allow() |  |
|  |  | Limit() |  |
|  |  |  |  |
| **Aspect-modifying predicates:** | | | |
|  | Completed() |  | marks that a process ends in a result state |
|  | Duration() |  | turns a process into an endeavor |
|  | Irrealis() |  | may be used to make result states optional |
|  | Repeated sequence() |  | turns a sequence of events into a process |
