# Supplementary material for: Semantic Representations for NLP Using VerbNet and the Generative Lexicon
Source: Front Artif Intell. 2022 Apr 14;5:821697. doi: 10.3389/frai.2022.821697 (PMC9048683; doi:10.3389/frai.2022.821697)
Supplement: Supplementary file 3 [file Table_3.DOCX]

**Appendix C: Semantic Clusters of Predicates**

**Causation**

cause()

in_reactio_to()

**Temporal**

**Duration**

spend_time()

duration()

**Schedule**

temporal_motion()

has_temporal_location()

**Sequence**

meets()

overlaps()

repeated_sequence()

co-temporal()

**Speed**

pace()

**Aspectual**

finish()

start()

completed()

irrealis()

repeated_sequence()

succeed()

**Authority dynamics**

yield()

charge()

authority_relationship()

subjugated()

**Body**

**Process**

sleep()

body_process()

body_reflex()

**Sensation**

discomfort()

body_sensation()

**Change**

**Value**

change_value()

**State**

become()

develop()

**Info**

declare()

transfer_info()

financial_interaction()

**Spatial**

**Location**

motion()

elliptical_motion()

emit()

**Posture**

elliptical_motion()

intrinsic_motion()

body_motion()

**Orientation**

rotational_motion()

**Possession**

transfer()

spend()

earn()

**Cognition**

has_information()

believe()

calculate()

think()

understand()

conclude()

**Adjudge**

judge()

assess()

characterize()

declare()

suspect()

**Perception**

perceive()

seem()

visible()

search()

appear()

find()

**Communication**

has_information()

transfer_info()

has_sentiment()

financial_interaction()

declare()

characterize()

declare()

**Information**

support()

has_information()

transfer_info()

has_sentiment()

think()

understand()

conclude()

**Harm/benefit**

harm()

benefit()

Desire

desire()

**Exist**

alive()

be()

endure()

**Financial**

financial_interaction()

financial_interest_in()

cost()

earn()

**Function**

act()

function()

**Harm** harm()

harmed()

endangered()

injury()

degradation_material_integrity()

destroyed()

suffocated()

**Intention**

succeed()

encourage()

discourage()

approve()

control()

abide_by()

ensure()

intend()

allow()

attempt()

dedicate()

**Location**

admit()

reside()

avoid()

confined()

appear()

free()

**Manner**

manner()

involuntary()

**Requirement**

necessitate()

require()

satisfy()

**Personal care**

take_care_of()

wear()

**Spatial/physical**

part_of()

contain()

covered()

full_of()

has_configuration()

has_location()

has_material_integrity_state()

has_position()

made_of()

penetrating()

has_capacity()

has_orientation()

has_physical_form()

attached()

mingled()

contact()

together()

fictive_motion()

has_boundary()

has_spatial_relationship()

support()

**Inclusion**

part_of()

has_set_member()

contain()

full_of()

has_capacity()

involved()

**Procreation**

give_birth()

procreate()

**Relationship**

equals()

correlated()

depend()

differ()

indicate()

signify()

harmonize()

relate()

exceed()

opposition()

support()

satisfy()

limit()

**Employment**

act()

has_role()

has_orgainzational_role()

authority_relationship()

work()

**Social**

cooperate()

conflict()

**States**

has_state()

**Result states**

adjusted()

cooked()

voided()

harmed()

endangered()

destroyed()

suffocated()

degradation_material_integrity()

confined()

covered()

involved()

free()

subjugated()

**Possession**

has_possession()

has_information()

**Attributes**

has_designation()

has_attribute()

has_sentiment()

has_emotional_state()

desire()

has_value()

cost()

about()

has_set_member()

has_spatial_relationship()

has_location()

has_boundary()

has_position()

has_orientation()

has_physical_form()

has_configuration()

penetrating()

support()

covered()

has_capacity()

contain()

full_of()

has_material_integrity_state()

attached()

mingled()

contact()

together()

made_of()

part_of()
